# Geographical Diversity of Proteomic Responses to Cold Stress in the Fungal Genus *Pseudogymnoascus*

**DOI:** 10.1007/s00248-023-02311-w

**Published:** 2023-12-07

**Authors:** Nurlizah Abu Bakar, Benjamin Yii Chung Lau, Marcelo González-Aravena, Jerzy Smykla, Beata Krzewicka, Saiful Anuar Karsani, Siti Aisyah Alias

**Affiliations:** 1 Institute of Ocean and Earth Sciences, C308, Institute of Advanced Studies Building, Universiti Malaya, 50603 Kuala Lumpur, Malaysia; 2 National Antarctic Research Centre, B303, Institute of Advanced Studies Building, Universiti Malaya, 50603 Kuala Lumpur, Malaysia; 3 Advanced Biotechnology and Breeding Centre, Malaysian Palm Oil Board, No. 6, Persiaran Institusi, Bandar Baru Bangi, 43000 Kajang, Selangor Malaysia; 4 Instituto Antártico Chileno, Plaza Muñoz Gamero, 1055 Punta Arenas, Chile; 5 Department of Biodiversity, Institute of Nature Conservation, Polish Academy of Sciences, Mickiewicza 33, 31-120 Krakow, Poland; 6 W. Szafer Institute of Botany, Polish Academy of Sciences, Lubicz 46, 31-512 Kraków, Poland; 7 Institute of Biological Sciences, Faculty of Science, Universiti Malaya, 50603 Kuala Lumpur, Malaysia

**Keywords:** Soil microfungi, Metabolic pathways, Cold adaptation, Lipid metabolism, Fungal adaptation, Methane metabolism

## Abstract

In understanding stress response mechanisms in fungi, cold stress has received less attention than heat stress. However, cold stress has shown its importance in various research fields. The following study examined the cold stress response of six *Pseudogymnoascus* spp. isolated from various biogeographical regions through a proteomic approach. In total, 2541 proteins were identified with high confidence. Gene Ontology enrichment analysis showed diversity in the cold stress response pathways for all six *Pseudogymnoascus* spp. isolates, with metabolic and translation-related processes being prominent in most isolates. 25.6% of the proteins with an increase in relative abundance were increased by more than 3.0-fold. There was no link between the geographical origin of the isolates and the cold stress response of *Pseudogymnoascus* spp. However, one Antarctic isolate, *sp3*, showed a distinctive cold stress response profile involving increased flavin/riboflavin biosynthesis and methane metabolism. This Antarctic isolate (*sp3*) was also the only one that showed decreased phospholipid metabolism in cold stress conditions. This work will improve our understanding of the mechanisms of cold stress response and adaptation in psychrotolerant soil microfungi, with specific attention to the fungal genus *Pseudogymnoascus*.

**Supplementary Information:**

The online version contains supplementary material available at 10.1007/s00248-023-02311-w.

## Introduction

Cold environments encompass many of the Earth’s biomes, including polar regions and alpine environments. Along with frequent and often long-lasting sub-zero temperatures, these environments are often characterized by frequent freeze–thaw cycles, high salt concentrations, low moisture and nutrient availability, and extreme ultraviolet (UV) and solar radiation. Despite the harshness, they are inhabited by a high diversity of biota, composed predominantly of microorganisms such as bacteria, protists, and fungi [13, 20].

Cold-adapted (psychrotolerant) fungi provide a large portion of low-temperature biodiversity and are essential for maintaining ecosystem processes in cold environments [20, 37]. However, while they grow at sub-zero temperatures, most can cope with wide temperature ranges and have a growth optimum above 15°C [20, 28, 36]. Fungi are also highly abundant and widespread in temperate zones and are even found in artificial habitats, such as refrigerated environments [20, 37]. For the genus *Pseudogymnoascus*, a multilocus phylogenetic analyses and morphological characterizations have determined four new species in Antarctica: *Pseudogymnoascus antarcticus* sp. nov., *Pseudogymnoascus australis* sp. nov., *Pseudogymnoascus griseus* sp. nov., and *Pseudogymnoascus lanuginosus* sp. nov. [35]. Moreover, new secondary metabolites have been described for these fungi isolated from Antarctica, demonstrating the recent interest in this group of organisms [19, 31].

Studies have demonstrated that the great efficiency of cold-adapted fungi and their ability to cope with extreme environmental conditions depends on various molecular and physiological adaptations, including the production of antifreeze proteins, compatible solutes (i.e., glycerol, trehalose, polyols) and cold-active enzymes [20, 34]. These findings are crucial from both evolutionary and biotechnological points of view [5, 17, 23]. Various cosmopolitan model organisms such as *Saccharomyces cerevisiae* Hansen and *Aspergillus nidulans* (Eidam) G. Winter are important model organisms of fungal stress tolerance. Nonetheless, psychrophilic and psychrotolerant fungi have also been studied to provide specific details and information on their cold-adapted properties. Improving understanding of the cold stress responses of psychrotolerant fungi is nevertheless an important research field. However, it should also be noted that the natural micro-environments of many fungi are often extremely variable [18]. Therefore, relatively stable experimentally-applied cold stress and non-stress conditions do not replicate the natural environmental variation [3]. Thus, caution must be applied when interpreting data from experimental laboratory studies. From a proteomic perspective, cold stress responses are expected to involve a balanced production of protein networks within cells to eliminate the damaging effects of low-temperature stress while sustaining normal cell processes. Various mechanisms are proposed to underlie the overall complexity of fungal cold stress responses [15, 20]. Many of the proposed mechanisms involve a range of cold-adapted metabolic pathways [16, 22] and translation-related processes [10, 14]. For instance, the cold stress responses of *Aspergillus flavus* Link and *Exophiala dermatitidis* (Kano) de Hoog showed increased activity of metabolic pathways involved in amino acid and carbohydrate metabolism [4, 33]. In *Flammulina velutipes* (Curtis) Singer and *Umbelopsis isabellina* (Oudem.) Gams, the upregulation of energy metabolism pathways, and ATP production were reported [21, 27]. Various lipid metabolic pathways are also involved in the cold stress response of fungi, including the metabolism of sphingolipids, phospholipids, and unsaturated fatty acids [15, 32]. Lipid modulation is significantly related to the stability of fungal membrane structures and their integrity, allowing survival after freezing [26, 29]. The cold stress response of fungi also includes various translation-related processes, such as the upregulation of SRP-dependent co-translational protein targeting membrane pathway, different cold-adapted ribosomal protein biosynthesis, and translation elongation pathways [4, 14, 32].

Progressive advances in proteomic technologies have enhanced our understanding and biotechnological application of psychrotolerant fungi [1, 27]. For instance, some fungal cold stress response mechanisms are applicable in producing antibiotics, antifungal molecules, secondary metabolites, and methane metabolism [25, 38]. However, their potential biotechnological application value is still underexplored and unrecognized. Considering these limitations, we aimed to investigate the cold stress response mechanisms of *Pseudogymnoascus* spp. using a proteomic approach. To elucidate potential broad-scale differences in cold stress response mechanisms, isolates from three different and geographically distant regions were selected, including polar regions (i.e., Arctic and Antarctic) and Europe as a temperate region. Our findings provide important baseline data on cold stress responses of soil microfungi that are needed to enhance further research on their biotechnological applications.

## Methodology

### Fungal Cultivation and Cold Stress Experimental Design

Four isolates of *Pseudogymnoascus* spp. from the polar regions, including the Arctic (HND16 R4-1 sp.1 and HND16 R2-1 sp.2) and Antarctic (AK07KGI1202 R1-1 sp.3 and AK07KGI1202 R1-1 sp.4) were obtained from the culture collection of the National Antarctic Research Centre (NARC), Universiti Malaya, Malaysia. Isolation, identification, and phylogenetic analysis of these isolates are described in a prior publication from our group [39]. Phylogenetic analysis clustered all isolates within an undescribed group of *Pseudogymnoascus* sequences; thus, they were described as *Pseudogymnoascus sp*. [39]. All the isolates were kept in pure culture, and their sequences were deposited into the GenBank database. Two isolates of *Pseudogymnoascus pannorum* (Link) Minnis & D.L. Lindner (CBS 106.13 and CBS 107.65) that originated from the temperate region (Switzerland and Germany) were purchased from the Westerdijk Fungal Biodiversity Institute which was previously known as Centraalbureau voor Schimmelcultures (CBS-KNAW) Fungal Biodiversity Centre (Utrecht, The Netherlands). The list of the investigated isolates with information on their origin and identification codes is given in Table 1. Fungal colony plugs (ca. 5 mm in diameter) were inoculated onto 100 mm Petri dishes of Czapek-Dox agar (CDA, Oxoid) and incubated in cold stress (CS) conditions (5 °C) and at optimal temperature (15 °C) for control (C) for 5 days. In this work, 5°C and an incubation period of 5 days were chosen to represent cold stress conditions for all isolates. Our previous work showed a clear indication of stress-related changes in colony morphology at 5°C, and no growth was observed below 5°C for all isolates. [2]. The incubation period was fixed at 5 days to ensure that all fungi cells were maintained in log phase growth since no significant difference in growth rates between day 5 and day 7 for all six isolates was observed [2].

**Table 1:** Pseudogymnoascus spp. isolates used in this study with information on their origin and identification codes

*Taxon name*	*Isolate code*	*Code used in text*	*Region*	*Sampling location*	*Coordinates*	*GenBank accession number*	*Collection*
*Pseudogymnoascus* sp*.*	HND16 R4-1 sp.1	*sp1*	Arctic	Hornsund, Spitsbergen	77°00′04″N, 15°33′37″E)	MK443476	NARC, Malaysia
*Pseudogymnoascus* sp*.*	HND16 R2-1 sp.2	*sp2*	Arctic	Hornsund, Spitsbergen	77°00′04″N, 15°33′37″E)	MK443477	NARC, Malaysia
*Pseudogymnoascus* sp*.*	AK07KGI1202 R1-1 sp.3	*sp3*	Antarctic	Fildes Peninsula, King George Island	62°12′57″S, 058°57′35 ″W	MK443474	NARC, Malaysia
*Pseudogymnoascus* sp*.*	AK07KGI1202 R1-1 sp.4	*sp4*	Antarctic	Fildes Peninsula, King George Island	62°12′57″S, 058°57′35″W	MK443475	NARC, Malaysia
*P. pannorum*	CBS 106.13	*C106*	Temperate	Sainte-Croix, near Yverdon, Switzerland	n.a	MH854616	Westerdijk Fungal Biodiversity Institute
*P. pannorum*	CBS 107.65	*C107*	Temperate	Schleswig–Holstein, Kiel-Kitzeberg, Germany	n.a	MH858505	Westerdijk Fungal Biodiversity Institute

*n.a: data not available, detailed information can be accessed from Westerdijk Fungal Biodiversity Institute website

### Preparation of Protein Extracts

Mycelia of *Pseudogymnoascus* spp. (from 10-day cultures) were carefully scraped using a sterile spatula. An average of 1 g of fungi mycelia (initial wet mass) was inoculated into 300 mL of Czapek-Dox liquid cultures in three replicates and grown for 5 days at the selected experimental temperatures (i.e., 5 °C and 15 °C). After 5 days, fungal biomass was harvested using a 0.45-µm filter paper and transferred to sterile tubes for weighing. Then, the harvested biomass was immediately flash-frozen and ground into fine powder in liquid nitrogen. Further steps of protein extract preparation were carried out following Tesei et al. [33] with modifications. Briefly, 1 g of ground mycelia was incubated in lysis buffer (7 M urea, 2 M thiourea, 4% CHAPS, 30 mM tris HCl, pH 8.5) for 1 h. The mixture was bath-sonicated for 15 min at 20 °C. Then 5 mL of tris-buffered phenol solution pH 8.0 (Sigma Aldrich) was added to the cell lysate, and the phenolic phase was collected after centrifugation (3300 × g for 20 min). Proteins were precipitated overnight at -20 °C by adding 5 volumes of 20% (w/v) ice-cold TCA/acetone (with the addition of 0.2% DTT, w/v). After centrifugation at 10,000 × g for 30 min, the precipitate was washed twice with ice-cold acetone (80%, v/v). The resulting pellet was air-dried and resuspended in 100 µl of modified lysis buffer (2 M urea, 30 mM tris HCl, pH 8.5). Total protein content was determined using a standard Bradford protein assay [8].

### In-solution Protein Digestion

In-solution protein digestion was carried out following Lau and Othman [24]. The extracted proteins (50 µg) were suspended in 100 µL of 50 mM ammonium bicarbonate and 1 M urea. The proteins were reduced and alkylated using 100 mM tris(2 carboxyethyl)phosphine and 200 mM iodoacetamide. Sodium deoxycholate in 5 mM ammonium bicarbonate [1% (w/v)] was added to the reduced and alkylated proteins to enhance the tryptic digestion at 37 °C for 10 min. Tryptic digestion using 1 µg of sequencing grade trypsin (Promega, Madison, WI, USA) per 50 µg protein was performed at 37 °C for 17 h. The resulting peptide mixture was then acidified with 0.5% formic acid to precipitate sodium deoxycholate through centrifugation at 14,000 × g (Eppendorf, Thermo Scientific) at ambient room temperature for 15 min. The remaining solvents and acids were removed using a centrifugal evaporator (CentriVap Concentrator, Labconco, MO, USA). The desiccated peptides were suspended in 100 µL of 0.1% formic acid and gently mixed before peptide purification. An Empore™ solid phase extraction disk (3 M Purification, Inc., MN, USA), conditioned with acetonitrile and methanol, was added into the peptide solution and incubated at the ambient temperature for 3 h to bind the peptides. Elution of the peptides from the disk was done twice using 50% ACN in 0.1% FA for 30 min each.

### Liquid Chromatography Tandem Mass Spectrometry Analysis (LC-MS/MS)

Peptides were reconstituted in 30 µL of 0.1% FA and 5% ACN. Then, 2 µL of the digest was loaded onto an Acclaim PepMap 100 C18 column (3 µm, 0.075 × 150 mm) (Thermo Scientific, MA, USA). The reverse phase column was equilibrated with 0.1% FA (mobile phase A) and 80% ACN in 0.1% FA (mobile phase B). A gradient of 5–35% mobile phase B over 70 min, at a flow rate of 300 nL min^−1^, was applied to elute the peptides. The peptides were separated using the EASY-nano liquid chromatography (EASY-nLC) 1200 System (Thermo Scientific, MA, USA). An online Q Exactive Plus Hybrid Quadrupole-Orbitrap mass spectrometer system (Thermo Scientific, MA, USA) generated the peptide ions with a spray voltage of 1800 V in positive mode. The precursor ion scan was conducted with a resolution of 70,000 and a mass range of *m/z* 310–1800. Precursors containing charge states from 2 + to 8 + were fragmented further. The fragmentation was done via collision-induced and high-energy collision-induced at a normalized energy of 28%. The resolution, isolation window, and ion injection time were set at 17,500, 0.7 Da, and 60 ms, respectively. The scanned precursor mass range was set at *m/z* 110–1800.

### Protein Identification and Bioinformatic Analysis

Mass spectra of the peptides were acquired using Xcalibur (Ver. 4.1.31.9) (Thermo Scientific, MA, USA) and deconvoluted with Proteome Discoverer (Ver. 2.4) (Thermo Scientific, MA, USA) to create the peptide mass list. SEQUEST HT search engine, incorporated in the Proteome Discoverer, was used to match the generated mass list against *Pseudogymnoascus destructans* (Blehert & Gargas) Minnis & D.L. Lindner (Taxonomy ID is 655981, 82,900 sequences). Mass tolerance for the proteins and their fragments was fixed at 10 ppm and 0.02 Da, respectively. Trypsin was indicated as the digestion enzyme, with up to two missed cleavages allowed during the search. Carbamidomethylation modification on cysteine residues was set as a static modification, whereas variable amino acid modifications included deamidation (asparagine and glutamine residues) and oxidation (methionine residues). The mass list was also searched against a decoy database generated from randomized protein sequences of the taxonomy mentioned earlier. Only proteins having at least the Rank 1 peptide and a false discovery rate of 1% were accepted. Spectra that matched the sequences were further validated using the Percolator algorithm (Ver. 2.04) with *q*-value at 1% false discovery rate. Venn diagrams were generated using the web-based Venny v2.1 software available at https://bioinfogp.cnb.csic.es/tools/venny/index.html (Oliveros 2007–2015).

### Peptide Quantification and Bioinformatics Analysis

The protein function was determined by inputting protein identifiers (NCBI accession number) into the UniProtKB database (http://www.uniprot.org/blast/) and assigning the respective Gene Ontology (GO) terms and annotations. Protein abundance values were used to calculate each isolate’s log_2_ ratios of CS:C for each isolate. A microarray (MA) plot was constructed using log_2_ CS:C ratios against -log_10_ local false discovery rate (FDR) values. A cut-off value of 1% FDR was applied to all data obtained from LC–MS/MS and quantification before performing MA plot analysis. Relative abundances (RA) were identified from the protein abundance data with a minimum of ± 0.1-fold change. The proteins that were significantly increased and decreased in relative abundance were determined with a 1.5-fold change as the cut-off value. Venn diagrams were also constructed to compare RAs of isolates within regions.

Differences in protein abundances between cold stress (CS) and control (C) conditions were analyzed by label-free relative quantitation method with the Proteome Discoverer v2.4 software. Briefly, the following parameters were used: precursor quantitation was based on intensity; normalization mode and scaling mode were set as “total peptide amount” and “on all average.” Protein abundances and ratios were calculated using the summed abundances and pairwise ratios. In this method, protein ratios were calculated as the median of all possible pairwise peptide ratios between the replicates of all related peptides. These values were normalized by the sum of their abundances for each channel over all peptides identified.

### Gene Ontology Enrichment Analysis

KOBAS v2.0 (http://kobas.cbi.pku.edu.cn) was used to search for gene enrichment. This software uses gene-level statistics called overrepresentation analysis [40]. The analysis is based on the hypergeometric distribution/Fisher’s exact test with the addition of Benjamini and Hochberg [7] false discovery rate (FDR) correction. FASTA sequences were used to identify enriched pathways in the KEGG, BioCyc, and Reactome databases based on changes in protein abundances between CS and C conditions. GO terms with p ≤ 0.05 were considered significantly enriched. *Saccharomyces cerevisiae* was selected as the reference Ascomycota species.

## Results

### Response of Proteomic Profiles to Cold Stress

**Fig. 1:**
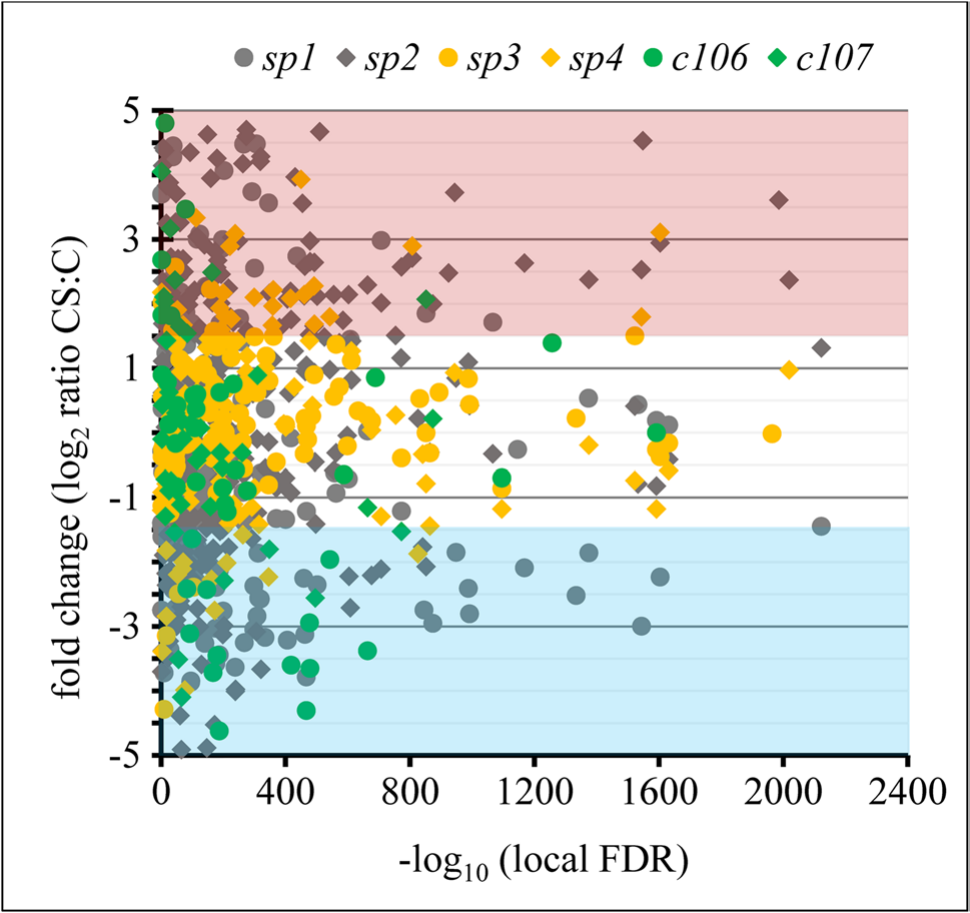
The microarray analysis (MA) plot showing the distribution of 2,541 proteomic profiles of proteins identified in all six isolates of *Pseudogymnoascus* spp. under cold stress (CS). Proteins that pass a threshold of 1.5-fold change were determined as significantly up- or downregulated (red- and blue-shaded area, respectively). Colors and shapes represent different isolates; Arctic: *sp1* – grey circle, *sp2* – grey diamond, Antarctic: *sp3* – yellow circle, *sp4* – yellow diamond, and temperate region: *C106* – green circle, *C107* – green diamond

The intracellular protein extracts from *Pseudogymnoascus* spp. isolates were analyzed using tandem liquid chromatography-mass spectrometry (LCMS/MS). A total of 2541 proteins were identified with high confidence (*p* < 0.01) from all six isolates in cold stress (CS) and control (C) conditions (Supplementary 1). The shift in the distribution of protein abundances under CS was demonstrated on a microarray analysis (MA) plot (Fig. 1). The fold change ratios of increased or decreased relative protein abundance (RA) with a minimum of ± 0.1-fold were plotted against -log_10_ local false discovery rate (FDR). The MA analysis identified 720 RA in all six isolates, with relatively similar proportions being increased and decreased in RA; 383 (53.2%) and 337 (46.8%) proteins, respectively. The majority of identified proteins (i.e., 71.7%) were clustered close to 0 and had relatively high confidence values (− log_10_ FDR > 800). All isolates showed a similar distribution pattern of RA under CS with no indication of differences related to geographical origin.

**Fig. 2:**
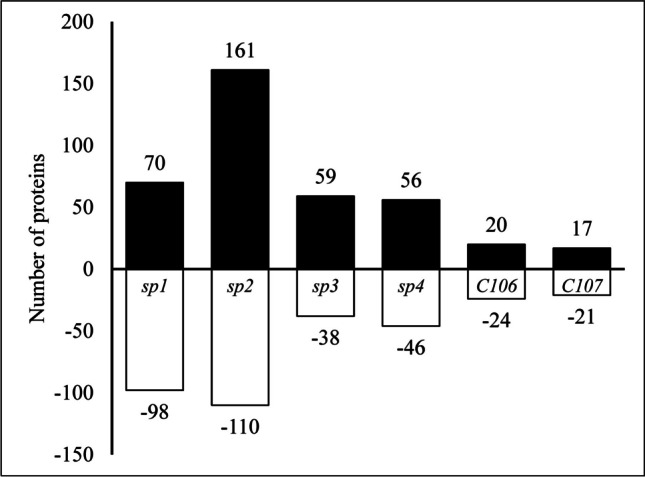
Bar graph showing the number of differentially expressed proteins (DEPs) in response to cold stress (CS) in isolates of *Pseudogymnoascus* spp. from different geographical regions. Values by the bars represent the number of differentially expressed proteins (DEPs); + values, upregulated proteins; – values, downregulated proteins. The Arctic isolates: *sp1*, *sp2*; Antarctic isolates: *sp3*, *sp4*; temperate isolates: *C106*, *C107*

A stacked bar graph was constructed to better visualize the distribution patterns of RA for each isolate (Fig. 2). The total number of RA differed noticeably among isolates from different geographical regions, with the highest numbers for the Arctic (168–271), intermediate for the Antarctic (97–102), and the lowest for the temperate region (38–44). This pattern seems to be consistent for both increased and decreased RA. However, due to the high variation, no clear pattern was visible in the proportion of increased and decreased RA. For instance, one of the Arctic isolates (i.e., *sp2*) exhibited a considerably higher number of increased than decreased RA proteins: 161 (59.4%) vs. 110 (40.6%), respectively. The other Arctic isolate (i.e., *sp1*) provided the opposite proportion, with only 70 (41.7%) vs. 98 (58.3%) increased and decreased RA proteins, respectively. The numbers of decreased RA proteins in both Arctic isolates were relatively similar, so these reverse proportions were mostly due to the very high difference in number of increased RA proteins. On the other hand, both Antarctic isolates (i.e., *sp3* and *sp4*) produced very similar numbers and proportions of increased and decreased RA proteins, i.e., 59 (69.8%) vs. 38 (39.2%) for sp3 and 56 (54.9%) vs. 46 (45.1%) for sp4, respectively. Similarly, the temperate isolates (i.e., *C106* and *C107*) did not differ significantly in the number and proportion of increased and decreased RA proteins. Although, some proteins demonstrated the opposite pattern compared to the Antarctic isolates, with 20 (45.5%) vs. 24 (54.5%) for *C106* and 17 (44.7%) vs. 21 (55.3%) for *C106* increased and decreased RA proteins, respectively.

**Fig. 3:**
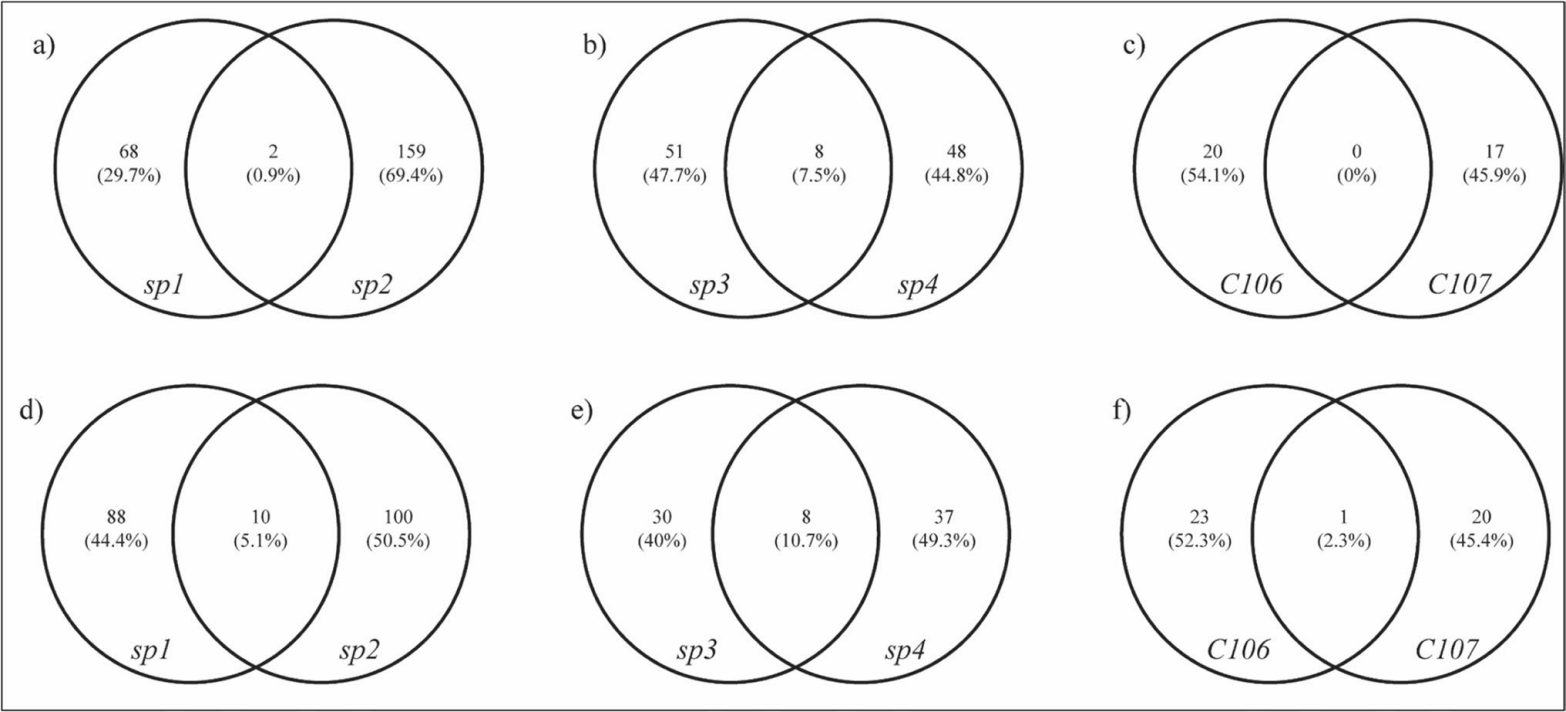
Venn diagrams showing the relationship between common and unique proteins in isolates from the same geographical region. **a**–**c** Upregulated proteins, **d**–**f** downregulated proteins. **a** and **d** the Arctic isolates, **b** and **e** the Antarctic isolates, **c** and **f** the temperate isolates

Simple Venn diagrams were used to illustrate the numbers of shared and unique proteins found in isolates from the same geographical region (Fig. 3). This analysis is crucial to show the degree of similarity or differences among isolates within regions to understand the relationship of RA proteins between isolates and to identify common proteins that potentially play a major role during cold stress in *Pseudogymnoascus* spp. The two Arctic isolates shared only two increased RA proteins (Fig. 3a), representing hypothetical proteins with molecular weights (MW) below 30 kDa. On the other hand, the Arctic isolates shared as much as 10 decreased RA proteins (Fig. 3d). These proteins were a mixture of enzymes (pyruvate dehydrogenase complex dihydrolipoamide acetyltransferase, ATP synthase F1, and isocitrate dehydrogenase), small subunit ribosomal proteins (S3e, S13, S20, and S22), structural protein (tubulin alpha-β chain), transporter protein (protein transporter sec-23) and degradation component protein (proteasome core particle subunit alpha 2). The Antarctic isolates shared 16 RA proteins, including eight increased and eight decreased RA proteins (Fig. 3b, e). Three of the shared RA proteins among increased RA proteins belonged to hypothetical proteins with MW over 30 kDa, and only one (GI number 1040529802) hypothetical protein VE03_04039 with a MW equal to 21.9 kDa. The other four of the eight shared RA proteins among increased RA proteins included translation initiation factor eIF4, guanine nucleotide-binding protein subunit beta-like protein, isocitrate dehydrogenase, and 60S ribosomal protein L20. The decreased RA proteins shared by the Antarctic isolates included NADP-specific glutamate dehydrogenase, mitochondrial heat shock protein 60, glucose-regulated protein, and 60S ribosomal protein L11. Surprisingly, the temperate isolates did not share any increased RA protein and had in common only one decreased RA protein, i.e., the plasma membrane ATPase (Fig. 3c, f).

### Gene Ontology Enrichment Analysis of Proteins Significantly Increased in Abundance (Fold Change of ≥ 1.5)

The significantly increased RA of *Pseudogymnoascus* spp. was further analyzed to identify significantly increased RA proteins with a fold change of ≥ 1.5 (Table 2). A total of 176 proteins were significantly increased in abundance across all isolates, with 47.7% (84) of them identified as hypothetical proteins. Among all the analyzed isolates, Arctic *sp2* had the highest numbers of significantly increased RA proteins, 91. Whereas temperate *C106* had the lowest numbers of significantly increased RA proteins, 5. In general, all *Pseudogymnoascus* spp. isolates significantly increased the abundance of various species of large ribosomal subunit proteins (i.e., L4e, L10a, L12, L16, L20, L21e, L26e, L35, and P0) and enzymes (e.g., catalase, pyruvate carboxylase, malate dehydrogenase, fatty acid synthase, transketolase, aldehyde dehydrogenase, enolase). Furthermore, 45 (25.6%) proteins were highly abundant with a fold change of ≥ 3.0. Surprisingly, only the Arctic *sp2* and Antarctic *sp4* isolates showed a significant increase in abundance of heat shock proteins and hsp-like protein species (i.e., heat shock protein SSB1 and hsp70-like proteins).

**Table 2:** List of significantly upregulated proteins under cold stress (fold change, log_2_ ratios of ≥ 1.5) as recorded in Supplementary 1

Isolate	Accession no. protein identified	Description of protein identified	Species of origin	Coverage [%]	# Unique peptides	# AAs	MW [kDa]	calc. pI	log_2_
*sp1*	1,352,887,886	Porin por1	*Pseudogymnoascus verrucosus*	94	6	283	30.3	8.98	1.9
	1,040,536,136	Hypothetical protein VF21_08556	*Pseudogymnoascus* sp. 05NY08	33	3	529	56	6.19	3.0
	1,040,511,155	Hypothetical protein VE04_08019	*Pseudogymnoascus* sp. 24MN13	16	6	400	42.1	6.15	2.6
	1,040,523,417	60S ribosomal protein L16	*Pseudogymnoascus* sp. 23,342–1-I1	37	1	202	23.1	10.51	2.2
	1,040,533,161	Catalase/peroxidase HPI	*Pseudogymnoascus* sp. 23,342–1-I1	23	5	795	87.3	5.5	3.0
	1,040,528,549	Large subunit ribosomal protein L26e	*Pseudogymnoascus* sp. 23,342–1-I1	41	11	137	15.7	10.68	1.9
	1,040,533,064	20S proteasome subunit alpha 7	*Pseudogymnoascus* sp. 23,342–1-I1	43	12	295	31.7	4.91	1.7
	1,040,498,553	Hypothetical protein VE00_08699	*Pseudogymnoascus* sp. WSF 3629	31	3	583	63.3	5.72	2.9
	1,040,563,505	Translocase of outer mitochondrial membrane	*Pseudogymnoascus verrucosus*	48	1	356	38.5	6	1.8
	1,040,504,933	Hypothetical protein VE00_02597	*Pseudogymnoascus* sp. WSF 3629	35	4	323	34.1	5.87	2.7
	1,352,887,735	Hypothetical protein VE01_04516	*Pseudogymnoascus verrucosus*	13	4	541	60.3	7.08	1.9
	1,040,528,536	Hypothetical protein VE03_05557	*Pseudogymnoascus* sp. 23,342–1-I1	65	7	580	63.1	6.52	1.7
	1,040,532,023	Hypothetical protein VE03_01299	*Pseudogymnoascus* sp. 23,342–1-I1	21	8	1015	105.6	5.01	3.6
	1,040,505,794	Hypothetical protein VE00_03008	*Pseudogymnoascus* sp. WSF 3629	28	8	186	21.4	11.18	2.1
	1,069,466,243	Large subunit ribosomal protein L21e	*Pseudogymnoascus verrucosus*	52	12	160	18.2	10.33	1.9
	1,040,526,528	Hypothetical protein VE03_07310	*Pseudogymnoascus* sp. 23,342–1-I1	25	12	1473	165.1	5.54	2.6
	1,040,525,391	Hypothetical protein VE03_07986	*Pseudogymnoascus* sp. 23,342–1-I1	21	6	783	81.9	5.11	4.5
	1,026,905,242	Hypothetical protein VC83_04831	*Pseudogymnoascus destructans*	14	2	1231	128.4	5.34	3.0
	1,040,534,050	Catalase/peroxidase HPI	*Pseudogymnoascus* sp. 05NY08	26	4	790	86.4	6.01	3.7
	1,040,525,523	Hypothetical protein VE03_08478	*Pseudogymnoascus* sp. 23,342–1-I1	25	5	790	87.1	5.85	4.5
	440,636,110	Hypothetical protein GMDG_07740	*Pseudogymnoascus destructans* 20,631–21	17	3	507	54	5.9	2.5
	1,040,502,501	Hypothetical protein VE00_05878	*Pseudogymnoascus* sp. WSF 3629	20	3	988	105.7	6.46	2.3
	1,040,530,925	Hypothetical protein VE03_02548	*Pseudogymnoascus* sp. 23,342–1-I1	14	5	1009	110.2	5.43	1.7
	1,040,547,240	Hypothetical protein VE02_07770	*Pseudogymnoascus* sp. 03VT05	8	3	486	51.9	6.11	1.6
	1,040,530,942	Hypothetical protein VE03_02444	*Pseudogymnoascus* sp. 23,342–1-I1	8	1	347	36.1	5.81	2.3
	1,040,504,056	Hypothetical protein VE00_03867	*Pseudogymnoascus* sp. WSF 3629	11	3	611	64.1	5.33	4.1
	1,040,520,853	Hypothetical protein VE04_00056	*Pseudogymnoascus* sp. 24MN13	31	3	505	54.2	8.24	3.1
	1,040,525,910	Hypothetical protein VE03_07646	*Pseudogymnoascus* sp. 23,342–1-I1	21	7	441	48.7	7.3	2.5
	1,069,466,961	Hypothetical protein VE01_02450	*Pseudogymnoascus verrucosus*	2	4	2096	226.9	4.91	2.3
	1,040,544,250	Proteasome subunit beta type-2	*Pseudogymnoascus* sp. 05NY08	19	2	182	20.4	7.49	1.7
	1,040,525,682	Hypothetical protein VE03_07697	*Pseudogymnoascus* sp. 23,342–1-I1	7	1	611	66.7	6.2	4.3
	1,040,520,567	Hypothetical protein VE04_00626	*Pseudogymnoascus* sp. 24MN13	9	4	646	71.5	7.3	4.5
	1,026,903,545	Mitochondrial import inner membrane translocase subunit tim8	*Pseudogymnoascus destructans*	60	4	89	10.1	6.05	1.6
	1,026,909,249	DASH complex subunit ask1	*Pseudogymnoascus destructans*	2	1	398	43.8	5.41	3.7
	1,040,507,167	Hypothetical protein VE00_00603	*Pseudogymnoascus* sp. WSF 3629	34	2	108	11.8	5.74	2.3
	1,040,503,472	Hypothetical protein VE00_03602	*Pseudogymnoascus* sp. WSF 3629	6	1	497	54	6.06	4.4
	1,040,531,469	Hypothetical protein VE03_02830	*Pseudogymnoascus* sp. 23,342–1-I1	7	3	713	76.9	6.49	2.0
*sp2*	1,040,547,996	ATP synthase subunit beta, mitochondrial	*Pseudogymnoascus* sp. 03VT05	87	3	516	55.4	5.68	2.4
	1,040,531,119	Glyceraldehyde 3-phosphate-dehydrogenase	*Pseudogymnoascus* sp. 23,342–1-I1	86	3	339	36.5	6.95	2.9
	1,040,553,812	Heat shock protein SSB1	*Pseudogymnoascus* sp. 03VT05	48	2	767	84.1	8.43	2.5
	1,040,537,109	ATP synthase subunit alpha, mitochondrial	*Pseudogymnoascus* sp. 05NY08	64	4	555	59.7	9.1	2.5
	1,040,529,266	Hypothetical protein VE03_04296	*Pseudogymnoascus* sp. 23,342–1-I1	13	4	4080	451.6	6.43	2.0
	1,040,523,711	hsp70-like protein	*Pseudogymnoascus* sp. 23,342–1-I1	65	10	676	73.5	5.74	2.4
	440,639,856	Tubulin beta chain	*Pseudogymnoascus destructans* 20,631–21	76	37	446	49.6	4.93	2.6
	1,040,524,485	Pyruvate carboxylase	*Pseudogymnoascus* sp. 23,342–1-I1	47	3	1190	130.3	6.35	1.5
	1,040,506,608	Actin	*Pseudogymnoascus* sp. WSF 3629	77	29	375	41.5	5.69	2.6
	1,026,904,149	Malate dehydrogenase, cytoplasmic	*Pseudogymnoascus destructans*	80	6	339	35.2	8.92	3.7
	1,040,560,294	Translation initiation factor eIF4A	*Pseudogymnoascus verrucosus*	67	26	398	44.9	5.24	1.7
	1,040,532,273	Ketol-acid reductoisomerase, mitochondrial	*Pseudogymnoascus* sp. 23,342–1-I1	69	2	400	44.5	7.05	2.1
	1,352,888,949	Phosphatidylinositol transfer protein csr1	*Pseudogymnoascus verrucosus*	63	1	221	24	9.39	3.0
	1,040,528,425	Hypothetical protein VE03_04962	*Pseudogymnoascus* sp. 23,342–1-I1	23	6	514	53.8	4.94	4.6
	1,040,530,832	Hypothetical protein VE03_02453	*Pseudogymnoascus* sp. 23,342–1-I1	45	6	462	48.7	8.29	2.6
	1,040,529,726	Cell division control protein 48	*Pseudogymnoascus* sp. 23,342–1-I1	60	53	823	89.9	5.05	2.7
	1,040,518,845	Fatty acid synthase subunit alpha	*Pseudogymnoascus* sp. 24MN13	30	1	1790	196.3	6.05	2.1
	1,040,530,016	Mitochondrial-processing peptidase subunit beta	*Pseudogymnoascus* sp. 23,342–1-I1	62	27	478	52.5	5.74	2.0
	1,040,501,521	Heat shock protein SSB1	*Pseudogymnoascus* sp. WSF 3629	62	5	614	66.5	5.44	4.5
	1,040,531,120	NADH-ubiquinone oxidoreductase 78 kDa subunit, mitochondrial	*Pseudogymnoascus* sp. 23,342–1-I1	59	4	741	80.6	6.57	2.1
	1,040,529,066	Hypothetical protein VE03_05085	*Pseudogymnoascus* sp. 23,342–1-I1	66	28	441	50.4	6.71	2.7
	1,040,530,740	Hypothetical protein VE03_03497	*Pseudogymnoascus* sp. 23,342–1-I1	51	13	572	60.8	5.26	1.6
	1,040,542,063	Hypothetical protein VF21_01051	*Pseudogymnoascus* sp. 05NY08	55	2	327	34.3	8.32	1.6
	1,026,906,053	erg10, acetyl-CoA C-acetyltransferase	*Pseudogymnoascus destructans*	79	5	399	41.2	6.8	2.1
	1,040,531,100	Plasma membrane ATPase	*Pseudogymnoascus* sp. 23,342–1-I1	41	2	931	100.8	5.15	2.3
	1,040,525,605	60S ribosomal protein L12	*Pseudogymnoascus* sp. 23,342–1-I1	57	2	165	17.7	9.33	2.2
	1,040,499,942	Hypothetical protein VE00_08276	*Pseudogymnoascus* sp. WSF 3629	26	8	473	49.1	5.82	2.7
	1,040,524,717	Transketolase	*Pseudogymnoascus* sp. 23,342–1-I1	35	7	685	74.8	5.97	2.6
	1,040,511,267	Hypothetical protein VE04_09537	*Pseudogymnoascus* sp. 24MN13	11	6	1822	202	6.47	2.4
	1,040,529,249	hypothetical protein VE03_04396	*Pseudogymnoascus* sp. 23,342–1-I1	65	2	468	51.3	5.54	2.2
	1,040,525,568	Hypothetical protein VE03_07513	*Pseudogymnoascus* sp. 23,342–1-I1	66	2	365	39.1	6.14	2.0
	1,069,466,751	Saccharopine dehydrogenase	*Pseudogymnoascus verrucosus*	47	3	503	55.2	5.71	2.5
	1,040,533,483	Hypothetical protein VE03_00182	*Pseudogymnoascus* sp. 23,342–1-I1	51	3	253	27.4	7.12	2.0
	1,040,528,274	Diphosphomevalonate decarboxylase	*Pseudogymnoascus* sp. 23,342–1-I1	48	16	385	40.8	6.55	2.8
	1,040,506,765	Glycine hydroxymethyltransferase	*Pseudogymnoascus* sp. WSF 3629	42	0	539	58.9	8.56	3.0
	1,040,529,880	Hypothetical protein VE03_04169	*Pseudogymnoascus* sp. 23,342–1-I1	30	10	1085	118.8	4.63	3.0
	1,040,505,261	Succinate dehydrogenase flavoprotein subunit, mitochondrial	*Pseudogymnoascus* sp. WSF 3629	60	26	646	70.8	6.49	1.8
	1,040,506,012	Hypothetical protein VE00_01435	*Pseudogymnoascus* sp. WSF 3629	16	25	2127	234.2	6.46	1.7
	1,040,500,818	Aldehyde dehydrogenase	*Pseudogymnoascus* sp. WSF 3629	67	1	496	53.4	5.95	1.7
	440,634,311	Catalase	*Pseudogymnoascus destructans* 20,631–21	60	2	505	57.4	7.3	1.5
	1,040,517,350	2,3-Bisphosphoglycerate-independent phosphoglycerate mutase	*Pseudogymnoascus* sp. 24MN13	49	8	522	57.7	5.4	2.2
	1,026,902,306	Hypothetical protein VC83_09257	*Pseudogymnoascus destructans*	50	4	545	60.5	6.11	4.3
	1,040,527,086	N-Acetyl-gamma-glutamyl-phosphate reductase/acetylglutamate kinase	*Pseudogymnoascus* sp. 23,342–1-I1	27	2	880	96.3	7.17	4.3
	1,040,503,795	Hypothetical protein VE00_03509	*Pseudogymnoascus* sp. WSF 3629	51	2	317	34.9	5.36	2.3
	1,040,538,418	Clathrin, heavy polypeptide	*Pseudogymnoascus* sp. 05NY08	15	1	1682	190.1	5.4	2.5
	1,040,543,888	T-complex protein 1 subunit gamma	*Pseudogymnoascus* sp. 05NY08	17	1	541	59	5.99	3.7
	1,040,504,412	Hypothetical protein VE00_02312	*Pseudogymnoascus* sp. WSF 3629	60	5	282	31.3	4.45	4.2
	1,040,531,360	Hypothetical protein VE03_02918	*Pseudogymnoascus* sp. 23,342–1-I1	32	6	210	22.8	4.87	2.0
	1,352,888,607	Target of Sbf	*Pseudogymnoascus verrucosus*	12	1	448	46.1	5.5	4.4
	1,040,530,118	Hypothetical protein VE03_04536	*Pseudogymnoascus* sp. 23,342–1-I1	19	9	542	60.3	7.61	3.3
	1,040,526,016	Plasma-membrane proton-efflux P-type ATPase	*Pseudogymnoascus* sp. 23,342–1-I1	13	2	990	108.4	5.39	2.1
	1,040,526,348	Dihydroxy-acid dehydratase	*Pseudogymnoascus* sp. 23,342–1-I1	32	8	592	63.2	7.12	2.9
	1,040,524,212	Hypothetical protein VE03_09540	*Pseudogymnoascus* sp. 23,342–1-I1	27	14	820	86.1	5.15	3.9
	1,040,501,074	Hypothetical protein VE00_07280	*Pseudogymnoascus* sp. WSF 3629	43	5	313	32.6	6.16	4.7
	1,040,501,360	40S ribosomal protein S17	*Pseudogymnoascus* sp. WSF 3629	39	3	148	17	9.8	4.6
	1,040,515,629	Hypothetical protein VE04_03766	*Pseudogymnoascus* sp. 24MN13	51	5	403	44.9	5.35	4.2
	1,040,501,480	Acetyl-CoA C-acetyltransferase	*Pseudogymnoascus* sp. WSF 3629	62	2	399	41.2	6.8	2.2
	1,069,464,671	Guanine nucleotide-binding protein subunit beta	*Pseudogymnoascus verrucosus*	55	3	355	39.1	7.4	1.5
	1,352,886,849	Proteasome regulatory particle base subunit rpt5	*Pseudogymnoascus verrucosus*	47	16	462	51.6	5.01	2.2
	1,040,504,856	ATP synthase F1, delta subunit	*Pseudogymnoascus* sp. WSF 3629	33	4	273	29	9.67	1.6
	1,040,532,080	26S protease regulatory subunit 6B	*Pseudogymnoascus* sp. 23,342–1-I1	38	2	421	47.1	6	2.7
	1,040,499,252	hsp70-like protein	*Pseudogymnoascus* sp. WSF 3629	63	4	682	73.9	5.19	3.6
	1,040,538,336	Hypothetical protein VF21_06756	*Pseudogymnoascus* sp. 05NY08	8	2	443	48.4	5.85	2.6
	440,640,697	Hypothetical protein GMDG_04885	*Pseudogymnoascus destructans* 20,631–21	10	2	666	70.3	5.26	2.7
	1,040,529,285	Hypothetical protein VE03_04902	*Pseudogymnoascus* sp. 23,342–1-I1	31	4	198	21.3	6.79	3.9
	1,040,548,610	Hypothetical protein VE02_07270	*Pseudogymnoascus* sp. 03VT05	6	2	577	64	6.79	3.8
	1,040,530,835	Hypothetical protein VE03_02559	*Pseudogymnoascus* sp. 23,342–1-I1	4	2	581	63.1	4.78	4.4
	440,637,926	Hypothetical protein GMDG_00466	*Pseudogymnoascus destructans* 20,631–21	18	2	334	35.7	5.6	1.5
	1,040,540,357	Hypothetical protein VF21_04798	*Pseudogymnoascus* sp. 05NY08	9	1	468	49.6	5.95	4.4
	1,040,526,301	Arginase	*Pseudogymnoascus* sp. 23,342–1-I1	13	2	330	35.4	5.62	2.2
	1,040,520,684	Hypothetical protein VE04_00111	*Pseudogymnoascus* sp. 24MN13	41	4	462	48.6	8.9	1.8
	1,040,547,997	NADH-ubiquinone oxidoreductase 78 kDa subunit, mitochondrial	*Pseudogymnoascus* sp. 03VT05	56	1	741	80.6	6.57	4.0
	1,040,529,431	Hypothetical protein VE03_03597	*Pseudogymnoascus* sp. 23,342–1-I1	3	1	309	32.8	5.66	2.7
	1,026,904,985	Hypothetical protein VC83_06014	*Pseudogymnoascus destructans*	22	2	239	26.1	5.62	2.7
	1,040,526,507	Hypothetical protein VE03_07003	*Pseudogymnoascus* sp. 23,342–1-I1	21	2	433	46.4	6.57	3.3
	1,040,518,253	Hypothetical protein VE04_03452	*Pseudogymnoascus* sp. 24MN13	9	2	282	31.3	5.54	2.2
	1,001,844,792	Pyridoxal biosynthesis lyase pdxS	Streptomyces *albidoflavus*	4	1	306	32.1	5.33	2.4
	1,040,539,921	Hypothetical protein VF21_03606	*Pseudogymnoascus* sp. 05NY08	13	2	415	46.8	5.41	2.5
	1,001,842,424	Inositol-1-phosphate synthase	Streptomyces *albidoflavus*	3	1	360	39.5	5.11	1.7
	1,040,532,999	Hypothetical protein VE03_00521	*Pseudogymnoascus* sp. 23,342–1-I1	14	4	469	50.7	5.36	2.1
	1,040,553,304	Hypothetical protein VE02_01894	*Pseudogymnoascus* sp. 03VT05	9	2	319	34.6	5.99	1.7
	1,040,502,810	Hypothetical protein VE00_05825	*Pseudogymnoascus* sp. WSF 3629	20	3	811	91.3	6.51	2.6
	1,040,564,605	Vacuolar protein 8	*Pseudogymnoascus verrucosus*	9	3	557	60.6	4.97	1.9
	1,040,539,273	Catalase	*Pseudogymnoascus* sp. 05NY08	54	1	505	57.3	7.47	4.7
	1,040,527,824	Catalase	*Pseudogymnoascus* sp. 23,342–1-I1	56	2	505	57.2	7.08	3.6
	1,040,541,436	Hypothetical protein VF21_04301	*Pseudogymnoascus* sp. 05NY08	44	1	250	27.3	6.32	1.5
	1,370,888,902	Hypothetical protein VC83_07881	*Pseudogymnoascus destructans*	80	2	330	34.1	6.39	2.1
	1,352,888,836	Protein disulfide-isomerase erp38	*Pseudogymnoascus verrucosus*	37	1	432	47.3	7.88	3.0
	1,069,467,799	Hypothetical protein VE01_03600	*Pseudogymnoascus verrucosus*	4	1	1126	123.7	6.21	1.6
	1,040,537,170	Urease accessory protein	*Pseudogymnoascus* sp. 05NY08	7	2	273	28.9	5.9	4.1
	1,040,532,480	GTP-binding protein rho2	*Pseudogymnoascus* sp. 23,342–1-I1	12	2	201	22.3	5.87	3.2
*sp3*	1,040,506,877	Enolase	*Pseudogymnoascus* sp. WSF 3629	85	43	438	47.7	5.41	1.5
	1,040,525,877	60S acidic ribosomal protein P0	*Pseudogymnoascus* sp. 23,342–1-I1	33	10	312	33.4	5.15	2.2
	1,040,513,597	20S proteasome subunit alpha 4	*Pseudogymnoascus* sp. 24MN13	55	12	267	29.2	7.4	1.6
	1,352,886,940	Hypothetical protein VE01_00604	*Pseudogymnoascus verrucosus*	20	3	249	26.2	6.61	1.6
	1,026,907,433	Cytochrome b-c1 complex subunit 7	*Pseudogymnoascus destructans*	25	4	123	14.4	9.07	2.6
	1,352,887,810	Hypothetical protein VE01_04771	*Pseudogymnoascus verrucosus*	8	3	329	36.1	6.33	2.0
	1,040,527,667	Hypothetical protein VE03_05893	*Pseudogymnoascus* sp. 23,342–1-I1	3	1	351	38.8	9.17	2.0
*sp4*	1,040,531,119	Glyceraldehyde 3-phosphate-dehydrogenase	*Pseudogymnoascus* sp. 23,342–1-I1	86	3	339	36.5	6.95	3.1
	1,040,553,812	Heat shock protein SSB1	*Pseudogymnoascus* sp. 03VT05	48	2	767	84.1	8.43	1.8
	1,040,560,294	Translation initiation factor eIF4A	*Pseudogymnoascus verrucosus*	67	26	398	44.9	5.24	2.3
	1,040,552,218	40S ribosomal protein S14	*Pseudogymnoascus* sp. 03VT05	79	12	150	16	10.87	2.2
	440,632,652	Large subunit ribosomal protein L4e	*Pseudogymnoascus destructans* 20,631–21	55	2	373	39.7	11.33	2.1
	1,040,529,726	Cell division control protein 48	*Pseudogymnoascus* sp. 23,342–1-I1	60	53	823	89.9	5.05	2.9
	1,040,526,037	Pyruvate kinase, variant	*Pseudogymnoascus* sp. 23,342–1-I1	55	5	562	61.1	7.72	1.8
	1,040,550,635	Small subunit ribosomal protein S2e	*Pseudogymnoascus* sp. 03VT05	55	17	273	29.2	10.27	2.9
	1,069,468,697	O-acetylhomoserine (thiol)-lyase	*Pseudogymnoascus verrucosus*	48	3	459	48.4	5.77	2.0
	1,069,465,551	Guanine nucleotide-binding protein subunit beta-like protein	*Pseudogymnoascus verrucosus*	66	3	316	35	7.03	2.2
	1,040,510,730	Hypothetical protein VE04_07723	*Pseudogymnoascus* sp. 24MN13	70	27	383	43.3	5.27	3.9
	1,040,560,461	Hypothetical protein VE01_06128	*Pseudogymnoascus verrucosus*	43	14	384	44.2	8.73	1.9
	1,026,906,053	erg10, acetyl-CoA C-acetyltransferase	*Pseudogymnoascus destructans*	79	5	399	41.2	6.8	2.1
	1,026,905,733	Hypothetical protein VC83_05243	*Pseudogymnoascus destructans*	39	5	169	18.7	4.67	1.7
	1,040,524,717	Transketolase	*Pseudogymnoascus* sp. 23,342–1-I1	35	7	685	74.8	5.97	1.7
	1,040,532,244	Hypothetical protein VE03_02026	*Pseudogymnoascus* sp. 23,342–1-I1	49	5	346	37	5.52	3.1
	1,040,529,249	Hypothetical protein VE03_04396	*Pseudogymnoascus* sp. 23,342–1-I1	65	2	468	51.3	5.54	2.1
	1,069,466,751	Saccharopine dehydrogenase	*Pseudogymnoascus verrucosus*	47	3	503	55.2	5.71	1.6
	1,040,502,157	Hypothetical protein VE00_04658	*Pseudogymnoascus* sp. WSF 3629	59	17	342	38.9	7.84	1.8
	1,040,529,736	Guanine nucleotide-binding protein subunit beta	*Pseudogymnoascus* sp. 23,342–1-I1	46	2	355	39.1	7.4	1.5
	440,638,868	GTP-binding protein ypt1	*Pseudogymnoascus destructans* 20,631–21	47	8	201	22.2	5.44	3.3
	1,370,882,703	Delta(24)-sterol C-methyltransferase	*Pseudogymnoascus destructans*	22	2	377	42.5	6.38	1.9
	1,069,464,671	Guanine nucleotide-binding protein subunit beta	*Pseudogymnoascus verrucosus*	55	3	355	39.1	7.4	1.8
	1,040,526,609	Hypothetical protein VE03_06110	*Pseudogymnoascus* sp. 23,342–1-I1	32	2	71	7.4	6.79	1.5
	1,040,499,366	Hypothetical protein VE00_09247	*Pseudogymnoascus* sp. WSF 3629	33	5	158	17.8	5.08	1.9
	1,026,909,249	DASH complex subunit ask1	*Pseudogymnoascus destructans*	2	1	398	43.8	5.41	2.2
	1,040,530,204	dTDP-glucose 4,6-dehydratase	*Pseudogymnoascus* sp. 23,342–1-I1	64	2	423	47.4	6.18	1.7
*C106*	1,026,908,689	60S ribosomal protein L35	*Pseudogymnoascus destructans*	46	1	125	14.4	11	1.8
	1,040,526,755	Hypothetical protein VE03_06971	*Pseudogymnoascus* sp. 23,342–1-I1	3	3	2518	275.9	5.55	3.5
	1,040,537,179	Hypothetical protein VF21_06185	*Pseudogymnoascus* sp. 05NY08	1	1	1161	124	6.24	2.7
	1,001,843,575	Catalase	*Streptomyces albidoflavus*	3	1	487	55.8	5.57	4.8
	1,040,511,525	Malate synthase, glyoxysomal	*Pseudogymnoascus* sp. 24MN13	1	1	542	60.5	7.72	1.8
*C107*	1,352,887,886	Porin por1	*Pseudogymnoascus verrucosus*	94	6	283	30.3	8.98	2.1
	1,040,557,179	60S ribosomal protein L10a	*Pseudogymnoascus verrucosus*	47	11	218	24.2	9.83	2.5
	1,040,502,912	60S ribosomal protein L20	*Pseudogymnoascus* sp. WSF 3629	35	1	184	22	10.76	1.5
	1,040,515,250	Hypothetical protein VE04_06692	*Pseudogymnoascus* sp. 24MN13	7	1	752	80.7	5.73	1.6
	1,370,880,553	Hypothetical protein VC83_03778	*Pseudogymnoascus destructans*	15	4	233	26.1	9.8	2.1
	1,040,560,581	Hypothetical protein VE01_06723	*Pseudogymnoascus verrucosus*	8	2	289	31	8.73	2.0
	1,026,909,729	Hypothetical protein VC83_01760	*Pseudogymnoascus destructans*	23	4	228	25.7	7.09	2.4
	1,040,503,482	Hypothetical protein VE00_03591	*Pseudogymnoascus* sp. WSF 3629	1	1	517	56.3	8.68	4.1
	1,040,502,988	4-nitrophenyl phosphatase	*Pseudogymnoascus* sp. WSF 3629	10	4	306	33.4	5.31	3.2

**Fig. 4:**
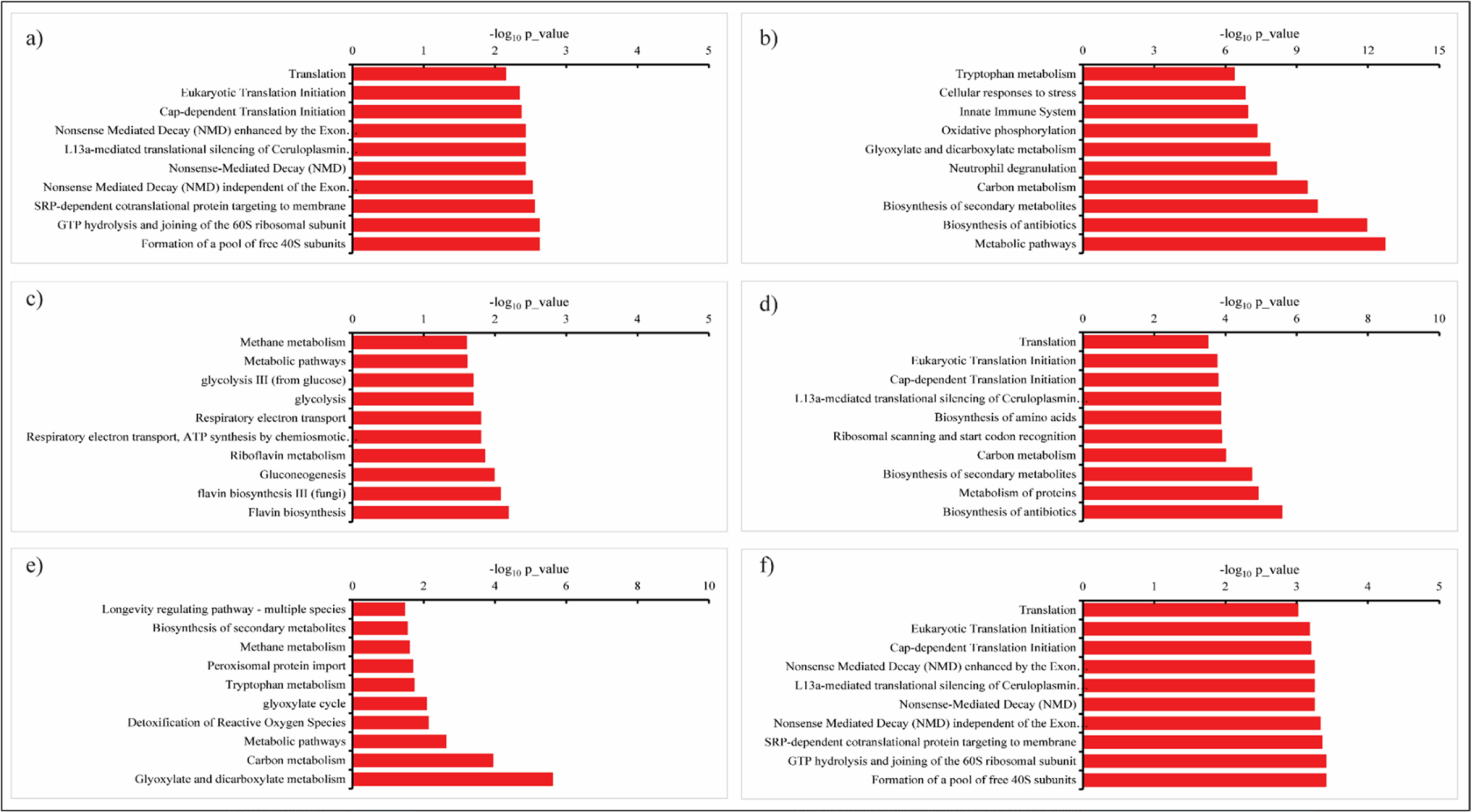
GO enrichment analysis of significantly upregulated proteins of *Pseudogymnoascus* spp. in response to cold stress (top 10 pathways). The Arctic isolates: **a** *sp1* and **b** *sp2*; Antarctic isolates **c** *sp3* and **d** *sp4*; and temperate isolates: **e** *C106* and **f** *C107*

GO enrichment analysis was carried out for all 176 significantly increased RA proteins in response to cold stress using KOBAS v2.0 to search for over-represented categories of molecular pathways in the databases Kyoto Encyclopedia of Genes and Genomes (KEGG), Panther, BioCyc, and Reactome. A complete list of enriched pathways with p values ≤ 0.05 for each isolate is given in Supplementary 2. The top 10 pathways and their respective p values for each isolate are presented in Fig. 4. Surprisingly, the increased RA proteins represented a high variety of pathways. Still, no common pathways were shared between pairs of isolates from the same geographical region. For instance, in the Arctic *sp1* and temperate *P. pannorum C107* isolates, the majority of enriched pathways were related to various translation processes, including the SRP-dependent co-translational protein targeting to membrane, cap-dependent translation initiation, eukaryotic translation initiation, various nonsense-mediated decay (NMD) processes, and ribosomal-related pathways (i.e., the formation of a pool of free 40S subunits, GTP hydrolysis and joining of the 60S ribosomal subunits) (Fig. 4a, f). On the other hand, in the Arctic *sp2* and temperate *P. pannorum C106* isolates, metabolic-related pathways were enriched, including tryptophan, carbon, glyoxylate, and dicarboxylate metabolism pathways, and biosynthesis of secondary metabolites (Fig. 4b, e). In addition, the Arctic *sp2* isolate showed enrichment of cellular responses to stress, biosynthesis of antibiotics, and activation of the innate immune system (Fig. 4b). In contrast, the temperate *P. pannorum C106* isolate also demonstrated enrichment of some additional pathways, such as methane metabolism, peroxisomal protein import, longevity regulating pathway and detoxification of reactive oxygen species (ROS) (Fig. 4e). A more distinct profile was observed in the Antarctic *sp3* isolate with the majority of enriched pathways related to energy production, such as the glycolysis, gluconeogenesis, and respiratory electron transport (ETC), and flavin/riboflavin metabolism pathways (Fig. 4c). However, the Arctic *sp3* isolate showed the same enriched methane metabolism pathway, as the temperate *P. pannorum C106* isolate. On the other hand, in the Antarctic *sp4* isolate, the enriched pathways showed similarities with both the Arctic and temperate isolates (Fig. 4d). The increased RA proteins in that isolate showed enrichment of various metabolic pathways, mainly the biosynthesis of secondary metabolites, antibiotics, and amino acids. Moreover, the Arctic *sp4* isolate also showed enrichment of protein and carbon metabolism pathways and various translation processes such as ribosomal scanning and start codon recognition, cap-dependent, and eukaryotic translation initiation pathways.

### Gene Ontology Enrichment Analysis of the Proteins Significantly Decreased in Abundance (with a Fold Change of ≥ −1.5)

Significantly decreased RA proteins (with a fold change of ≥  − 1.5) recorded in isolates of *Pseudogymnoascus* spp. in response to cold stress are listed in Table 3. A total of 148 proteins were significantly decreased in abundance across all six isolates, with 77 (52.0%) identified as hypothetical proteins. Forty-six (31.1%) of the proteins were decreased in abundance with a fold change of at least -3.0-fold. Arctic isolates (i.e., *sp1* and *sp2*) showed many significantly decreased RA proteins (54 and 55, respectively). In contrast, one of the Antarctic isolates (i.e., *sp3*) had the lowest number of significantly decreased RA proteins (5), all were identified as hypothetical proteins. In general, the significantly decreased RA proteins included various enzymes in energy production processes such the TCA cycle, glycolysis and gluconeogenesis, glyceraldehyde 3-phosphate-dehydrogenase, ATP-citrate synthase subunit 1, phosphoglycerate kinase, succinyl-CoA ligase subunit beta, isocitrate dehydrogenase, acetyl Co-A hydrolase, fatty acid synthase subunit beta, and pyruvate kinase. Heat shock proteins or hsp-like proteins were only significantly decreased in abundance in the Arctic *sp1* isolate (heat shock protein SSB1 and hsp70-like protein).

**Table 3:** List of significantly downregulated proteins under cold stress (fold change, log_2_ ratios of ≥ -1.5) recorded in Supplementary 1

Isolate	Accession no. protein identified	Description of protein identified	Species of origin	Coverage [%]	# Unique peptides	# AAs	MW [kDa]	calc. pI	log_2_
*sp1*	1,040,543,410	Elongation factor 1-alpha	*Pseudogymnoascus* sp. 05NY08	75	11	459	49.9	9.13	− 2.8
	1,040,531,119	Glyceraldehyde 3-phosphate-dehydrogenase	*Pseudogymnoascus* sp. 23,342–1-I1	86	3	339	36.5	6.95	− 2.2
	1,040,553,812	Heat shock protein SSB1	*Pseudogymnoascus* sp. 03VT05	48	2	767	84.1	8.43	− 3.0
	1,040,525,455	Hypothetical protein VE03_07380	*Pseudogymnoascus* sp. 23,342–1-I1	63	4	231	25.6	6.42	− 3.8
	1,040,502,460	Molecular chaperone HtpG	*Pseudogymnoascus* sp. WSF 3629	69	1	703	79.5	4.92	− 2.5
	1,040,529,266	Hypothetical protein VE03_04296	*Pseudogymnoascus* sp. 23,342–1-I1	13	4	4080	451.6	6.43	− 3.0
	1,040,523,711	hsp70-like protein	*Pseudogymnoascus* sp. 23,342–1-I1	65	10	676	73.5	5.74	− 1.9
	440,639,856	Tubulin beta chain	*Pseudogymnoascus destructans* 20,631–21	76	37	446	49.6	4.93	− 2.1
	1,040,499,891	Hypothetical protein VE00_07973	*Pseudogymnoascus* sp. WSF 3629	41	8	196	21.4	7.88	− 3.0
	1,040,528,567	ATP-citrate synthase subunit 1	*Pseudogymnoascus* sp. 23,342–1-I1	74	8	668	72.4	8.34	− 2.7
	1,040,540,892	Elongation factor EF-3	*Pseudogymnoascus* sp. 05NY08	58	3	1064	117.6	6.27	− 2.4
	1,040,531,987	Serine hydroxymethyltransferase, cytosolic	*Pseudogymnoascus* sp. 23,342–1-I1	51	1	484	53.3	7.78	− 3.1
	1,040,532,273	Ketol-acid reductoisomerase, mitochondrial	*Pseudogymnoascus* sp. 23,342–1-I1	69	2	400	44.5	7.05	− 3.2
	1,040,532,121	Fatty acid synthase subunit beta	*Pseudogymnoascus* sp. 23,342–1-I1	43	7	2109	233.4	5.72	− 1.8
	1,040,552,218	40S ribosomal protein S14	*Pseudogymnoascus* sp. 03VT05	79	12	150	16	10.87	− 2.8
	1,352,887,002	Hypothetical protein VE01_00786	*Pseudogymnoascus verrucosus*	41	1	292	31.6	8.92	− 2.3
	1,040,537,116	Argininosuccinate synthase	*Pseudogymnoascus* sp. 05NY08	58	5	416	46.4	5.48	− 2.8
	440,637,842	Phosphoglycerate kinase	*Pseudogymnoascus destructans* 20,631–21	62	1	417	44.4	6.47	− 2.3
	1,040,531,120	NADH-ubiquinone oxidoreductase 78 kDa subunit, mitochondrial	*Pseudogymnoascus* sp. 23,342–1-I1	59	4	741	80.6	6.57	− 2.4
	1,069,462,575	Small subunit ribosomal protein S12e	*Pseudogymnoascus verrucosus*	59	11	148	16.4	4.94	− 2.4
	1,040,548,810	Succinyl-CoA ligase subunit beta, mitochondrial	*Pseudogymnoascus* sp. 03VT05	51	4	445	47.9	5.48	− 2.6
	1,040,533,135	ATP synthase F1, gamma subunit	*Pseudogymnoascus* sp. 23,342–1-I1	48	6	298	32.1	8.34	− 3.3
	1,040,504,430	40S ribosomal protein S20	*Pseudogymnoascus* sp. WSF 3629	38	5	116	13.1	9.63	− 1.5
	1,040,527,945	Triosephosphate isomerase	*Pseudogymnoascus* sp. 23,342–1-I1	74	7	249	26.8	5.76	− 3.2
	1,040,525,856	Eukaryotic translation Initiation factor 3 subunit B	*Pseudogymnoascus* sp. 23,342–1-I1	30	1	744	84.5	5.01	− 3.6
	1,040,507,376	40S ribosomal protein S22	*Pseudogymnoascus* sp. WSF 3629	58	8	130	14.5	9.89	− 1.9
	440,631,821	Hypothetical protein GMDG_00116	*Pseudogymnoascus destructans* 20,631–21	56	1	231	25.4	4.48	− 3.4
	1,040,532,244	Hypothetical protein VE03_02026	*Pseudogymnoascus* sp. 23,342–1-I1	49	5	346	37	5.52	− 3.6
	1,026,905,771	Intracellular distribution of mitochondria	*Pseudogymnoascus destructans*	10	10	1291	142.5	5.72	− 1.5
	1,040,532,770	V-type proton ATPase subunit B	*Pseudogymnoascus* sp. 23,342–1-I1	60	24	516	57.5	5.74	− 3.0
	1,040,526,111	Glutamine synthetase	*Pseudogymnoascus* sp. 23,342–1-I1	64	19	366	40.6	5.8	− 1.9
	1,069,477,437	40S ribosomal protein S13	*Pseudogymnoascus verrucosus*	46	7	151	16.8	10.32	− 1.6
	1,040,504,427	T-complex protein 1, zeta subunit	*Pseudogymnoascus* sp. WSF 3629	29	12	541	59	6.49	− 1.8
	1,040,497,161	pyrABCN	*Pseudogymnoascus* sp. WSF 3629	10	4	2245	246.8	6	− 2.3
	1,040,506,854	Glutamine-fructose-6-phosphate transaminase	*Pseudogymnoascus* sp. WSF 3629	31	11	1087	120.8	6.49	− 2.4
	1,040,538,646	Isocitrate dehydrogenase, mitochondrial	*Pseudogymnoascus* sp. 05NY08	43	1	459	51.5	8.76	− 2.1
	1,040,545,371	Hypothetical protein VE02_09611	*Pseudogymnoascus* sp. 03VT05	25	10	510	56	9.31	− 3.1
	1,040,504,491	Hypothetical protein VE00_02345	*Pseudogymnoascus* sp. WSF 3629	34	1	357	39.5	5.49	− 2.2
	1,040,532,437	Acetolactate synthase I/II/III large subunit	*Pseudogymnoascus* sp. 23,342–1-I1	14	7	694	75.3	8.82	− 3.9
	1,040,564,667	Importin alpha subunit (Karyopherin alpha subunit) (Serine-rich RNA polymerase I suppressor protein)	*Pseudogymnoascus verrucosus*	25	11	552	60.2	5.11	− 3.3
	1,040,504,412	Hypothetical protein VE00_02312	*Pseudogymnoascus* sp. WSF 3629	60	5	282	31.3	4.45	− 2.6
	1,040,504,451	Hypothetical protein VE00_02372	*Pseudogymnoascus* sp. WSF 3629	4	1	271	29.1	6.55	− 1.6
	1,040,533,074	Hypothetical protein VE03_00632	*Pseudogymnoascus* sp. 23,342–1-I1	42	11	219	22.9	5.27	− 1.8
	1,040,506,454	Phosphoribosylamine-glycine ligase/phosphoribosylformylglycinamidine cyclo-ligase	*Pseudogymnoascus* sp. WSF 3629	23	3	785	83.7	5.4	− 2.1
	1,040,522,863	Hypothetical protein VE03_10908, partial	*Pseudogymnoascus* sp. 23,342–1-I1	9	3	1176	126.6	6.89	− 3.8
	1,040,528,502	Riboflavin synthase, alpha subunit	*Pseudogymnoascus* sp. 23,342–1-I1	19	3	230	24.4	4.93	− 3.4
	1,040,520,742	T-complex protein 1 subunit alpha	*Pseudogymnoascus* sp. 24MN13	31	10	568	61.8	6.64	− 2.0
	1,352,887,691	Hypothetical protein VE01_04967	*Pseudogymnoascus verrucosus*	15	6	415	44.5	7.11	− 1.5
	1,026,908,370	Hypothetical protein VC83_03291	*Pseudogymnoascus destructans*	3	1	246	28.3	5.97	− 2.7
	1,040,505,097	Hypothetical protein VE00_01915	*Pseudogymnoascus* sp. WSF 3629	13	1	496	51.6	5.6	− 2.7
	1,040,526,837	Nitrite reductase	*Pseudogymnoascus* sp. 23,342–1-I1	3	3	1111	123.1	6.62	− 3.7
	1,040,516,031	Hypothetical protein VE04_03877	*Pseudogymnoascus* sp. 24MN13	19	1	326	35.7	4.98	− 2.4
	1,040,533,107	Glutamine-fructose-6-phosphate transaminase	*Pseudogymnoascus* sp. 23,342–1-I1	27	2	706	78.3	6.43	− 2.3
	1,040,506,083	Orotidine 5′-phosphate decarboxylase	*Pseudogymnoascus* sp. WSF 3629	4	1	357	38.9	5.19	− 1.5
*sp2*	1,352,887,886	Porin por1	*Pseudogymnoascus verrucosus*	94	6	283	30.3	8.98	− 2.1
	1,040,506,096	Adenosylhomocysteinase	*Pseudogymnoascus* sp. WSF 3629	55	1	450	48.7	5.68	− 1.8
	1,040,501,747	Hypothetical protein VE00_06979	*Pseudogymnoascus* sp. WSF 3629	29	4	383	39.7	5.95	− 4.5
	1,040,547,813	Dihydrolipoyl dehydrogenase	*Pseudogymnoascus* sp. 03VT05	56	2	509	54	7.03	− 2.2
	1,040,536,136	Hypothetical protein VF21_08556	*Pseudogymnoascus* sp. 05NY08	33	3	529	56	6.19	− 2.1
	1,040,496,401	60S ribosomal protein L11	*Pseudogymnoascus* sp. WSF 3629	40	8	172	19.8	10.17	− 2.4
	1,040,530,310	Hypothetical protein VE03_03010	*Pseudogymnoascus* sp. 23,342–1-I1	31	5	294	31.5	6.64	− 3.0
	1,040,529,999	60S ribosomal protein	*Pseudogymnoascus* sp. 23,342–1-I1	57	6	109	11.8	9.95	− 1.7
	1,040,541,287	40S ribosomal protein S27	*Pseudogymnoascus* sp. 05NY08	27	3	82	8.8	9.26	− 1.9
	1,040,528,747	Hypothetical protein VE03_03290	*Pseudogymnoascus* sp. 23,342–1-I1	11	6	487	47.1	4.46	− 1.8
	440,640,338	Dihydrolipoyl dehydrogenase	*Pseudogymnoascus destructans* 20,631–21	54	2	509	54.1	6.89	− 2.7
	1,040,507,376	40S ribosomal protein S22	*Pseudogymnoascus* sp. WSF 3629	58	8	130	14.5	9.89	− 1.6
	1,040,539,732	Hypothetical protein VF21_05012	*Pseudogymnoascus* sp. 05NY08	12	2	568	58.4	5.34	− 3.6
	1,040,530,977	Hypothetical protein VE03_01549	*Pseudogymnoascus* sp. 23,342–1-I1	53	5	315	32.7	9.31	− 1.8
	1,069,477,643	Vacuolar protease A	*Pseudogymnoascus verrucosus*	49	2	395	42.9	5.02	− 2.2
	1,040,533,064	20S proteasome subunit alpha 7	*Pseudogymnoascus* sp. 23,342–1-I1	43	12	295	31.7	4.91	− 1.5
	1,040,536,470	Hypothetical protein VF21_07858	*Pseudogymnoascus* sp. 05NY08	36	1	613	68.4	6	− 3.7
	440,635,254	60S ribosomal protein L23	*Pseudogymnoascus destructans* 20,631–21	45	8	139	14.6	10.21	− 2.0
	1,040,517,192	Aspartate-semialdehyde dehydrogenase	*Pseudogymnoascus* sp. 24MN13	43	2	364	38.9	6.77	− 1.8
	1,040,513,597	20S proteasome subunit alpha 4	*Pseudogymnoascus* sp. 24MN13	55	12	267	29.2	7.4	− 1.8
	1,040,527,436	Large subunit ribosomal protein L7Ae	*Pseudogymnoascus* sp. 23,342–1-I1	52	18	264	29.3	10.29	− 2.0
	1,040,528,493	Hypothetical protein VE03_04938	*Pseudogymnoascus* sp. 23,342–1-I1	48	6	336	37.4	9.09	− 2.1
	1,040,556,291	Proteasome subunit YC7alpha/Y8 (protease yscE subunit 7)	*Pseudogymnoascus verrucosus*	67	2	254	28	6.38	− 1.9
	1,040,525,350	Hypothetical protein VE03_08618	*Pseudogymnoascus* sp. 23,342–1-I1	15	3	441	47.1	4.59	− 1.9
	1,040,519,485	Hypothetical protein VE04_01330	*Pseudogymnoascus* sp. 24MN13	25	3	598	64.8	5.9	− 3.1
	1,040,525,391	Hypothetical protein VE03_07986	*Pseudogymnoascus* sp. 23,342–1-I1	21	6	783	81.9	5.11	− 3.1
	1,026,905,242	Hypothetical protein VC83_04831	*Pseudogymnoascus destructans*	14	2	1231	128.4	5.34	− 2.7
	1,040,534,050	Catalase/peroxidase HPI	*Pseudogymnoascus* sp. 05NY08	26	4	790	86.4	6.01	− 1.6
	1,040,504,793	Acetyl-CoA hydrolase	*Pseudogymnoascus* sp. WSF 3629	8	3	528	58.6	6.58	− 1.9
	1,040,530,925	Hypothetical protein VE03_02548	*Pseudogymnoascus* sp. 23,342–1-I1	14	5	1009	110.2	5.43	− 2.3
	1,040,516,362	Hypothetical protein VE04_05577	*Pseudogymnoascus* sp. 24MN13	35	4	140	15.4	8.12	− 2.2
	1,040,510,845	Large subunit ribosomal protein L4e	*Pseudogymnoascus* sp. 24MN13	38	2	356	37.8	10.93	− 4.0
	1,040,547,240	Hypothetical protein VE02_07770	*Pseudogymnoascus* sp. 03VT05	8	3	486	51.9	6.11	− 1.8
	1,040,523,481	Hypothetical protein VE03_09986	*Pseudogymnoascus* sp. 23,342–1-I1	26	1	300	32.3	6.44	− 2.7
	1,040,504,056	Hypothetical protein VE00_03867	*Pseudogymnoascus* sp. WSF 3629	11	3	611	64.1	5.33	− 3.0
	1,040,535,243	Hypothetical protein VF21_09386	*Pseudogymnoascus* sp. 05NY08	15	1	324	34.5	6.07	− 3.2
	1,040,531,382	Hypothetical protein VE03_02803	*Pseudogymnoascus* sp. 23,342–1-I1	7	2	617	64.4	5.31	− 3.0
	1,040,539,900	Hypothetical protein VF21_03593	*Pseudogymnoascus* sp. 05NY08	9	1	116	13.1	11.09	− 3.4
	1,352,887,341	Hypothetical protein VE01_02784	*Pseudogymnoascus verrucosus*	47	8	211	23.5	9.55	− 1.6
	1,040,507,457	Hypothetical protein VE00_00106	*Pseudogymnoascus* sp. WSF 3629	1	1	799	85.4	6.64	− 3.7
	1,040,506,170	Hypothetical protein VE00_01618	*Pseudogymnoascus* sp. WSF 3629	4	4	1255	140.5	6.98	− 2.1
	1,352,885,447	Hypothetical protein VC83_06581	*Pseudogymnoascus destructans*	21	1	808	89.3	7.01	− 4.0
	1,040,526,014	Hypothetical protein VE03_07417	*Pseudogymnoascus* sp. 23,342–1-I1	9	1	650	69.1	5	− 4.9
	1,040,515,456	Glucosamine-phosphate N-acetyltransferase	*Pseudogymnoascus* sp. 24MN13	21	3	180	20.1	5.87	− 2.2
	1,040,528,090	Hypothetical protein VE03_04733	*Pseudogymnoascus* sp. 23,342–1-I1	3	2	945	99.8	10.14	− 2.4
	1,040,523,420	Hypothetical protein VE03_10013	*Pseudogymnoascus* sp. 23,342–1-I1	9	2	507	56.2	6.04	− 3.5
	1,040,529,367	Hypothetical protein VE03_04870	*Pseudogymnoascus* sp. 23,342–1-I1	21	5	485	52.8	5.6	− 4.9
	1,040,499,918	Hypothetical protein VE00_07980	*Pseudogymnoascus* sp. WSF 3629	11	4	209	23.2	5.24	− 2.3
	1,040,503,265	Hypothetical protein VE00_05135	*Pseudogymnoascus* sp. WSF 3629	19	2	132	14.7	5.15	− 1.6
	440,636,209	Hypothetical protein GMDG_02002	*Pseudogymnoascus destructans* 20,631–21	40	1	132	14.3	4.74	− 2.9
	1,370,887,271	Leucine aminopeptidase 1	*Pseudogymnoascus destructans*	14	1	434	47.7	5.54	− 1.8
	1,040,539,268	Hypothetical protein VF21_05590	*Pseudogymnoascus* sp. 05NY08	7	2	528	54.5	5.55	− 2.3
	1,040,503,305	hypothetical protein VE00_05156	*Pseudogymnoascus* sp. WSF 3629	4	1	757	83.9	5.58	− 1.8
	1,040,547,051	Hypothetical protein VE02_08445	*Pseudogymnoascus* sp. 03VT05	7	3	524	53.8	5.64	− 2.6
	1,040,535,582	Endoribonuclease L-PSP	*Pseudogymnoascus* sp. 05NY08	49	1	128	13.8	6	− 4.4
*sp3*	1,040,528,747	Hypothetical protein VE03_03290	*Pseudogymnoascus* sp. 23,342–1-I1	11	6	487	47.1	4.46	− 2.5
	1,040,530,957	Hypothetical protein VE03_01500	*Pseudogymnoascus* sp. 23,342–1-I1	17	3	632	68.7	5.11	− 2.4
	1,040,526,014	Hypothetical protein VE03_07417	*Pseudogymnoascus* sp. 23,342–1-I1	9	1	650	69.1	5	− 2.4
	1,370,880,003	Hypothetical protein VC83_03064	*Pseudogymnoascus destructans*	8	2	304	30.7	8.05	− 3.1
	1,040,527,834	Hypothetical protein VE03_06437	*Pseudogymnoascus* sp. 23,342–1-I1	2	2	727	77.8	6.99	− 4.3
*sp4*	1,040,501,747	Hypothetical protein VE00_06979	*Pseudogymnoascus* sp. WSF 3629	29	4	383	39.7	5.95	− 2.8
	1,040,554,290	Aminopeptidase 2	*Pseudogymnoascus* sp. 03VT05	56	2	891	99.5	5.4	− 1.9
	1,040,532,023	Hypothetical protein VE03_01299	*Pseudogymnoascus* sp. 23,342–1-I1	21	8	1015	105.6	5.01	− 2.2
	1,040,543,638	Hypothetical protein VF21_01105	*Pseudogymnoascus* sp. 05NY08	19	2	627	67.7	8.37	− 2.3
	1,040,530,062	Hypothetical protein VE03_04519	*Pseudogymnoascus* sp. 23,342–1-I1	26	8	707	77.2	5.31	− 2.0
	1,040,528,450	Hypothetical protein VE03_04986	*Pseudogymnoascus* sp. 23,342–1-I1	17	2	375	40.3	5.81	− 4.0
	1,370,872,825	Hypothetical protein VC83_00609	*Pseudogymnoascus destructans*	1	1	1423	161.5	7.93	− 3.4
	1,040,517,617	Hypothetical protein VE04_03781	*Pseudogymnoascus* sp. 24MN13	15	3	305	31.9	4.64	− 2.1
	1,040,536,276	Hypothetical protein VF21_08313	*Pseudogymnoascus* sp. 05NY08	7	3	575	60.8	4.83	− 2.2
	1,069,469,765	Hypothetical protein VE01_04555	*Pseudogymnoascus verrucosus*	31	1	159	18.8	6.96	− 2.0
	1,370,880,003	Hypothetical protein VC83_03064	*Pseudogymnoascus destructans*	8	2	304	30.7	8.05	− 1.8
	1,069,462,037	Hypothetical protein VE01_00458	*Pseudogymnoascus verrucosus*	33	1	462	48.6	8.75	− 1.6
	1,040,524,869	Hypothetical protein VE03_08655	*Pseudogymnoascus* sp. 23,342–1-I1	7	2	415	46.9	6.67	− 2.8
*C106*	1,040,525,455	Hypothetical protein VE03_07380	*Pseudogymnoascus* sp. 23,342–1-I1	63	4	231	25.6	6.42	− 4.3
	1,352,888,949	Phosphatidylinositol transfer protein csr1	*Pseudogymnoascus verrucosus*	63	1	221	24	9.39	− 3.7
	1,040,526,037	Pyruvate kinase, variant	*Pseudogymnoascus* sp. 23,342–1-I1	55	5	562	61.1	7.72	− 2.0
	1,040,531,100	Plasma membrane ATPase	*Pseudogymnoascus* sp. 23,342–1-I1	41	2	931	100.8	5.15	− 3.4
	1,040,533,135	ATP synthase F1, gamma subunit	*Pseudogymnoascus* sp. 23,342–1-I1	48	6	298	32.1	8.34	− 5.0
	1,040,526,437	Hypothetical protein VE03_08106	*Pseudogymnoascus* sp. 23,342–1-I1	14	9	707	74.5	7.33	− 2.4
	1,040,505,261	Succinate dehydrogenase flavoprotein subunit, mitochondrial	*Pseudogymnoascus* sp. WSF 3629	60	26	646	70.8	6.49	− 3.6
	440,634,311	Catalase	*Pseudogymnoascus destructans* 20,631–21	60	2	505	57.4	7.3	− 2.9
	1,040,527,086	N-acetyl-gamma-glutamyl-phosphate reductase/acetylglutamate kinase	*Pseudogymnoascus* sp. 23,342–1-I1	27	2	880	96.3	7.17	− 3.5
	1,040,531,151	Hypothetical protein VE03_02408	*Pseudogymnoascus* sp. 23,342–1-I1	56	9	103	11.4	11.36	− 3.1
	1,040,519,866	T-complex protein 1 subunit epsilon	*Pseudogymnoascus* sp. 24MN13	33	12	548	59.7	5.5	− 3.7
	1,040,504,839	Hypothetical protein VE00_01830	*Pseudogymnoascus* sp. WSF 3629	48	9	192	20.7	8.62	− 1.6
	1,040,541,679	Hypothetical protein VF21_02713	*Pseudogymnoascus* sp. 05NY08	51	1	323	34.1	6.58	− 4.6
	1,040,501,615	Hypothetical protein VE00_06167	*Pseudogymnoascus* sp. WSF 3629	33	2	408	42.4	7.75	− 2.4
*C107*	1,040,506,608	Actin	*Pseudogymnoascus* sp. WSF 3629	77	29	375	41.5	5.69	− 1.5
	1,370,880,945	Methionine-synthesizing 5-methyltetrahydropteroyltriglutamate–homocysteine methyltransferase	*Pseudogymnoascus destructans*	49	1	768	86.2	6.58	− 2.6
	1,040,517,350	2,3-Bisphosphoglycerate-independent phosphoglycerate mutase	*Pseudogymnoascus* sp. 24MN13	49	8	522	57.7	5.4	− 1.8
	1,040,530,081	Hypothetical protein VE03_04439	*Pseudogymnoascus* sp. 23,342–1-I1	11	4	1819	201.3	6.27	− 2.3
	1,040,538,684	Hypothetical protein VF21_06127	*Pseudogymnoascus* sp. 05NY08	17	1	474	49.9	8.06	− 1.5
	1,040,550,136	Hypothetical protein VE02_06045	*Pseudogymnoascus* sp. 03VT05	17	3	275	29.5	6.04	− 4.1
	1,040,524,038	Hypothetical protein VE03_09547	*Pseudogymnoascus* sp. 23,342–1-I1	15	1	559	61.4	6.57	− 3.5

**Fig. 5:**
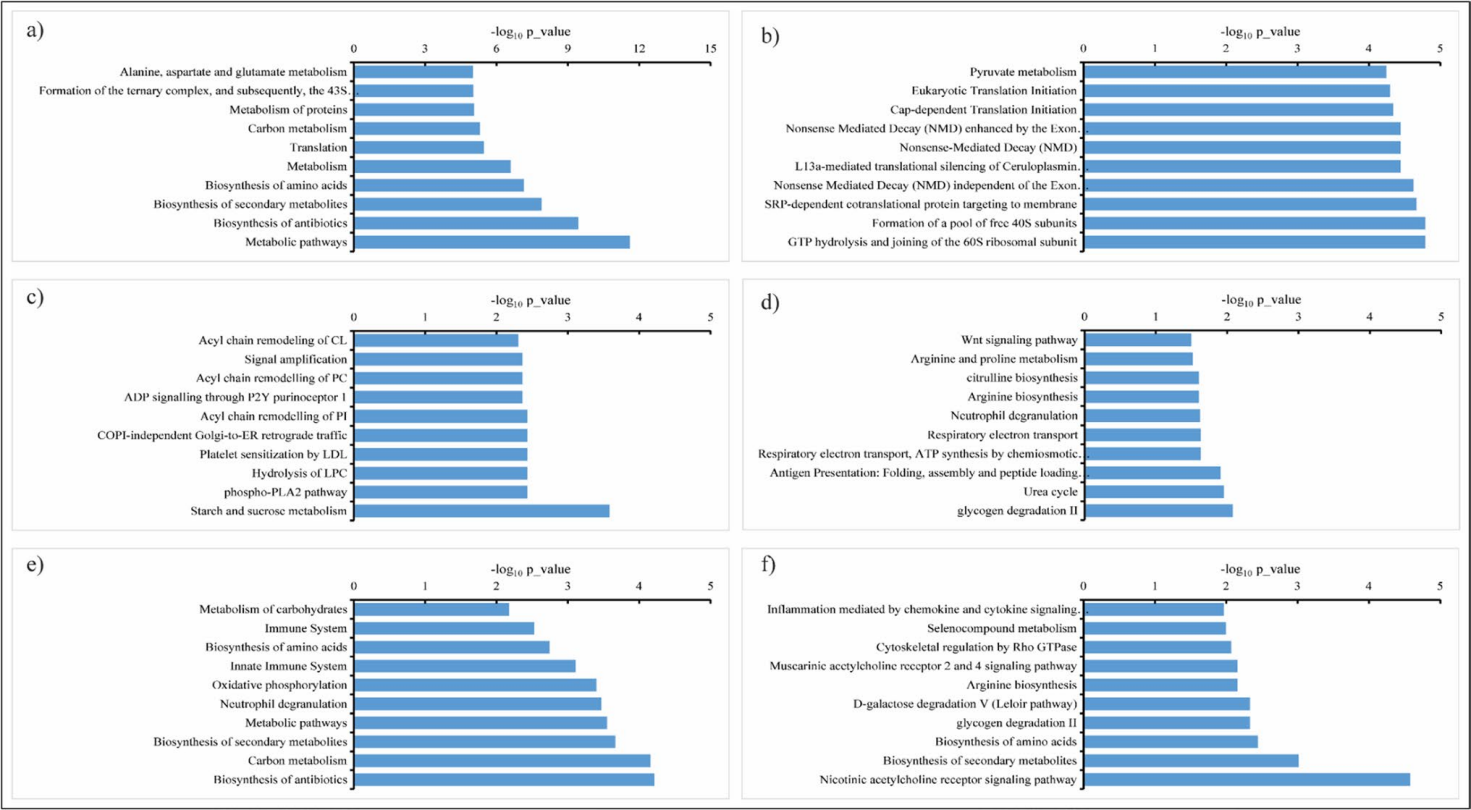
GO enrichment analysis of significantly downregulated proteins of *Pseudogymnoascus* spp. isolates in response to cold stress (the top 10 pathways). The Arctic isolates: **a** *sp1* and **b** *sp2*; Antarctic isolates: **c** *sp3* and **d** *sp4*; and temperate isolates: **e** *C106* and **f** *C107*

GO enrichment analysis of all 148 significantly decreased RA proteins showed a variety of metabolic and biosynthesis pathways enriched in all isolates (Fig. 5). Metabolic pathways related to protein homeostasis, such as protein metabolism and the biosynthesis of amino acids, were enriched in the Arctic *sp1*, Antarctic *sp4* and both temperate isolates (*C106* and *C107*) (Fig. 5a, d–f). The biosynthesis of secondary metabolites was also enriched in the Arctic *sp1* isolate and temperate isolates (*C106* and *C107*) (Fig. 5a, e–f). Carbon metabolism and biosynthesis of antibiotics were enriched in the Arctic *sp1* and temperate *C106* isolates (Fig. 5a, e), and glycogen degradation II in the Antarctic *sp4* and temperate *C107* isolate (Fig. 5d, f). The decreased protein pathways in the Antarctic *sp4* isolate also involved the wingless-related integration site (wnt) signaling pathway, neutrophil degranulation, respiratory electron transport (ETC), urea cycle, and activation of antigen pathway (Fig. 5d). In the Arctic *sp2* isolate, the majority of decreased protein pathways were related to translation processes such as the eukaryotic and cap-dependent translation initiation, nonsense-mediated decay (NMD) processes, and the formation of a pool of free 40S subunits (Fig. 5b). Pyruvate metabolism was also decreased in the Arctic *sp2* isolate. In the Antarctic *sp3* isolate, most decreased protein pathways were related to phospholipid metabolism involving pathways such as the phospho-PLA2, hydrolysis of lysophosphatidylcholine (LPC), acyl chain remodeling of cardiolipin (CL), phosphatidylcholine (PC), and phosphatidylinositol (PI). This isolate also showed downregulation of starch and sucrose metabolism and COPI-independent Golgi-to-ER retrograde traffic and signal amplification pathways (Fig. 5c).

## Discussion

### Variation in Proteomic Profiles of Pseudogymnoascus Spp. Isolates in Response to Cold Stress

All *Pseudogymnoascus* spp. isolates investigated in this work originated from environments that naturally experience cold temperatures, though they are exposed to wide variations in mean annual temperatures and distinct seasonal changes [6, 12]. Thus, all the investigated isolates from polar and temperate regions can be expected to share the same cold-adaptation mechanisms. However, it is worth noting that all six isolates are not of the same species, except for the temperate isolates (*C106* and *C107*), that belong to the species *P. pannorum* (Table 1). Our previous work on these isolates, when exposed to heat stress, showed a diversity of protein profiling with protein homeostasis, energy production, and DNA repair pathways being enriched [2].

Similar patterns of relative protein abundances in cold stress (CS) compared to control (C) conditions (log_2_ ratios CS:C) did not demonstrate any apparent geographical differences in cold stress responses among the investigated isolates (Fig. 1). The individual plots of RA for each isolate (Fig. 2) showed similar findings to the distribution patterns of RA as observed in the overall MA plot (Fig. 1), again with no indication of any effect from different geographical origins. However, variation in the total number of significantly increased and decreased RA and visible shifts in proportion between increased and decreased RA (Fig. 2) suggested some likely geographical differences in cold stress responses among *Pseudogymnoascus* spp. isolates. For instance, the temperate isolates had the lowest number of RA, and decreased RA proteins were more abundant than increased RA proteins. The Antarctic isolates had over twice the number of RA than the temperate isolates, and increased RA proteins were dominant. Then, the Arctic isolates provided the highest number of RA, but shifts in the proportion of increased and decreased RA were inconsistent. However, a very small number of shared RA (Fig. 3) indicates the existence of very high variation, even among isolates originating from the same geographical region. From our previous work on temperature-dependent growth analysis of all six isolates, the four polar isolates (*sp1*, *sp2*, *sp3*, and *sp4*) had an optimal growth temperature at 15°C and 25°C with no significant difference between the two temperatures [2]. For the temperate isolates (*C106* and *C107*), the optimal growth temperature was at 20°C. This suggests that the optimal growth temperature of isolates may contribute to the high number of increased and decreased RA proteins of all polar isolates compared to the temperate isolates exposed to cold stress. Therefore, analysis of a higher number of fungal isolates from all regions would be necessary to verify the consistency and significance of patterns apparent in our data.

The numbers of significantly increased and decreased RA proteins varied greatly between isolates, within a range of 5–91 proteins (Tables 2 and 3). It is noteworthy that 161 proteins were identified as hypothetical proteins from significantly increased and decreased RA proteins. These hypothetical proteins are important because they contribute to 49% of the overall significantly regulated proteins in all six isolates of *Pseudogymnoascus* spp. (161 from a total of 324 proteins). Our result suggests that *Pseudogymnoascus* spp. respond by altering only important proteins to preserve the lack of cumulative energy under cold stress. This is consistent with other studies, demonstrating a generally low number of differentially upregulated proteins. For instance, *Flammulina velutipes* (Curtis) Singer, a white-rot fungus that has a relatively low vegetative-growth temperature (20–24°C), under cold stress produced only 31 differentially upregulated proteins [27]. Likewise, a psychrophilic fungus *Mrakia psychrophila* M.X. Xin and P.J. Zhou showed increases in only 27 proteins when exposed to 4°C [32]. Similar findings were also reported for the mesophilic fungi *Mortierella isabellina* Oudem. M6-22 and *Exophiala dermatitidis* (Kano) de Hoog showed upregulation of only 29 and 33 proteins, respectively, when exposed to cold stress [21, 33]. However, while all previous studies investigated only single isolates, our work is the first to report the effects of cold stress on the proteome in several isolates of the same fungal genus from different geographical regions.

### Gene Ontology Enrichment Analysis

The composition of increased RA proteins in *Pseudogymnoascus* spp. exposed to cold stress indicated enrichment of various metabolic and translation-related pathways. This included mostly pathways involved in the metabolism of carbon, glyoxylate, dicarboxylate, methane, and amino acids. In addition, an increment was also observed for translation-related pathways, such as forming a pool of free 40S subunits, nonsense-mediated decay (NMD), and eukaryotic and cap-dependant translation initiation pathways. Surprisingly, no increment of an identical pathway was identified, even for isolates from the same geographical region. Response mechanisms to cold stress have already been investigated in several cosmopolitan and common fungi, including *Saccharomyces cerevisiae*, *Schizosaccharomyces pombe* Lindner, and *Aspergillus nidulans*. Studies of these “model” fungal species have resulted in the discovery of numerous stress-related proteins [9, 30]. These include various cold-adapted enzymes and protective molecules that are produced in cold-stress conditions to increase fungal cell stability [37].

However, only a limited number of studies reported on fungal cold stress response mechanisms with the application of proteomic profiling, and the majority of these focused on mesophilic fungi [21, 27, 33]. For instance, in *Exophiala dermatitidis*, cold stress-induced upregulation of the beta-oxidation of very long-chain fatty acids, glycolysis/gluconeogenesis, peroxisomal lipid metabolism, and cellular response to stress [33]. *Flammulina velutipes* also showed upregulation of amino acid biosynthesis, signaling pathways, and various energy metabolism pathways, such as the citrate cycle (TCA cycle), pentose phosphate pathway, glyoxylate, and dicarboxylate metabolism [27]. In comparison, the psychrophilic fungus *Mrakia psychrophila* demonstrated upregulation of energy metabolism and production of unsaturated fatty acids that regulate membrane fluidity [32]. In this work, we also showed a significant number of hypothetical proteins, demonstrating that functional studies on these proteins from polar fungi are still lacking. Hence, studying these functionally unknown sequences could provide additional insight into potential mechanisms governing cold adaptation of *Pseudogymnoascus* spp.

Previous studies have also demonstrated that low temperatures do not cause irreversible damage to fungal cells, and fungi respond to cold stress by modifying molecular content in their complex protein networks [27, 32]. Comparison across all six isolates of *Pseudogymnoascus* spp. studied here revealed no apparent geographical pattern in protein profiles or pathways involved. This was particularly the case for pathways of carbon metabolism, biosynthesis of amino acids, secondary metabolites and antibiotics, and translation-related pathways. Our findings suggest that *Pseudogymnoascus* spp. modulate various carbon and amino acid metabolism and translation-related pathways to minimize energy use for growth or cell division. We postulated that *Pseudogymnoascus* spp. adapt to cold stress by utilizing nutrient availability to support cell damage and repair and minimizing protein production for cell growth. Su et al. [32] reported that *Mrakia psychrophila* showed downregulation of TCA cycle, glycolysis, and ribosomal proteins. Similarly, *Exophiala dermatitidis* demonstrated downregulation of carbon and pyruvate metabolism and the pentose phosphate pathway [33]. In our study, only the Antarctic *sp3* isolate showed a distinctive profile of cold stress response. The upregulated pathways of that isolate included mainly flavin/riboflavin biosynthesis, glycolysis/gluconeogenesis, respiratory electron transport (ETC), and methane metabolism. It is important to indicate that biogenic methane production is generally only associated with prokaryotic microorganisms such as methanogens and Archaea [11]. However, Lenhart et al. [25] have suggested that terrestrial vegetation and fungi can also be involved in the production of methane. There are a few decreased protein pathways that were identified only in *sp3* and not in any others isolates, such as acyl chain remodeling of cardiolipin, phosphatidylcholine, phosphatidylinositol, and the hydrolysis of lysophosphatidylcholine. Phospholipids are key molecules involved in the maintenance of membrane fluidity and are also involved in signaling pathways. The metabolism of fungal phospholipids has been extensively studied in the model organism, *S. cerevisiae* [29].

## Conclusions

When exposed to cold stress, our results showed a variation in increased and decreased protein abundances between different isolates of *Pseudogymnoascus* spp. Several metabolic enzymes and ribosomal proteins were significantly increased and decreased in abundance in all six isolates of *Pseudogymnoascus* spp. examined. Pathway enrichment analysis also showed diversity in the cold stress response pathways, with metabolic and translation-related processes being prominent in most isolates. However, the Antarctic isolate *sp3* showed a distinctive cold stress response profile involving increased flavin/riboflavin biosynthesis and methane metabolism. The Antarctic *sp3* isolate is also the only one that showed decreased phospholipid metabolism when exposed to cold stress. Our results suggest that *Pseudogymnoascus* spp. adapt to cold stress by utilizing nutrient availability to support cell damage and repair with minimal need for cell growth. The cold stress response of the *Pseudogymnoascus* spp. isolates examined, while showing wide variation in the pathways enriched, did not show any obvious association with the biogeographical regions of origins of the isolates. The data obtained in this study provides new information on how *Pseudogymnoascus* spp. respond to temperature variations in their environments. This work also improves our understanding of their responses and adaptions toward varying environmental temperatures that may affect their survival in soil ecosystems. We would like to emphasize the need for whole genome analysis of *Pseudogymnoascus* spp. for future works to support functional annotation of unknown sequences of various hypothetical proteins, thus providing additional insight into potential mechanisms governing cold adaptation of *Pseudogymnoascus* spp.

### Supplementary Information

Below is the link to the electronic supplementary material.

Supplementary file1 (XLSX 10231 KB)

Supplementary file2 (XLS 167 KB)

## Data Availability

All data analyzed are provided in the manuscript and supplemental files. The raw datasets generated and analyzed during the study are available from the corresponding author upon reasonable request.

## Declarations

This study did not involve human subjects, and all reported work is original, and prevailing local, national, and international regulations and conventions and normal scientific ethical practices have been respected. Consent is given by all authors for publication in Microbial Ecology if accepted.
